# Development and application of G4-Flame as a visual biosensor for G4-DNA

**DOI:** 10.1093/nar/gkag179

**Published:** 2026-03-24

**Authors:** Ruyi Liu, Tao Wang, Chunxu Wang, Mingyou Xu, Boyu Chen, Jinyi Zhao, Wanxiang Xiong, Shixiang Pan, Zhao Ruan, Ningyuan Lu, Yuxin Zhang, Guang Yang, Fanzheng Meng, Yufeng Liu, Xuedan Sun, Lianxin Liu

**Affiliations:** Department of Hepatobiliary Surgery, State Key Laboratory of Immune Response and Immunotherapy, Centre for Leading Medicine and Advanced Technologies of IHM, The First Affiliated Hospital of USTC, Division of Life Sciences and Medicine, University of Science and Technology of China, Hefei, Anhui 230001, China; Anhui Provincial Key Laboratory of Hepatopancreatobiliary Surgery, Anhui Provincial Clinical Research Center for Hepatobiliary Diseases, Hefei, Anhui, China; School of Life Sciences, Division of Life Sciences and Medicine, University of Science and Technology of China, Hefei, Anhui 230001, China; High Magnetic Field Laboratory, Hefei Institutes of Physical Science, Chinese Academy of Sciences, Hefei 230031, China; Key Laboratory of Anhui Province for High Field Magnetic Resonance Imaging, Hefei 230031, China; Anhui Provincial High Magnetic Field Laboratory, Hefei 230031, China; Key Laboratory of High Magnetic Field and Ion Beam Physical Biology, Hefei Institutes of Physical Science, Chinese Academy of Sciences, Hefei 230031, China; Department of Hepatobiliary Surgery, State Key Laboratory of Immune Response and Immunotherapy, Centre for Leading Medicine and Advanced Technologies of IHM, The First Affiliated Hospital of USTC, Division of Life Sciences and Medicine, University of Science and Technology of China, Hefei, Anhui 230001, China; Anhui Provincial Key Laboratory of Hepatopancreatobiliary Surgery, Anhui Provincial Clinical Research Center for Hepatobiliary Diseases, Hefei, Anhui, China; School of Life Sciences, Division of Life Sciences and Medicine, University of Science and Technology of China, Hefei, Anhui 230001, China; School of Life Sciences, Division of Life Sciences and Medicine, University of Science and Technology of China, Hefei, Anhui 230001, China; Department of Hepatobiliary Surgery, State Key Laboratory of Immune Response and Immunotherapy, Centre for Leading Medicine and Advanced Technologies of IHM, The First Affiliated Hospital of USTC, Division of Life Sciences and Medicine, University of Science and Technology of China, Hefei, Anhui 230001, China; Anhui Provincial Key Laboratory of Hepatopancreatobiliary Surgery, Anhui Provincial Clinical Research Center for Hepatobiliary Diseases, Hefei, Anhui, China; School of Life Sciences, Division of Life Sciences and Medicine, University of Science and Technology of China, Hefei, Anhui 230001, China; School of Medicine, Anhui University of Science & Technology, Huainan 232001, China; School of Life Sciences, Division of Life Sciences and Medicine, University of Science and Technology of China, Hefei, Anhui 230001, China; Department of Hepatobiliary Surgery, State Key Laboratory of Immune Response and Immunotherapy, Centre for Leading Medicine and Advanced Technologies of IHM, The First Affiliated Hospital of USTC, Division of Life Sciences and Medicine, University of Science and Technology of China, Hefei, Anhui 230001, China; Anhui Provincial Key Laboratory of Hepatopancreatobiliary Surgery, Anhui Provincial Clinical Research Center for Hepatobiliary Diseases, Hefei, Anhui, China; School of Life Sciences, Division of Life Sciences and Medicine, University of Science and Technology of China, Hefei, Anhui 230001, China; Department of Hepatobiliary Surgery, State Key Laboratory of Immune Response and Immunotherapy, Centre for Leading Medicine and Advanced Technologies of IHM, The First Affiliated Hospital of USTC, Division of Life Sciences and Medicine, University of Science and Technology of China, Hefei, Anhui 230001, China; Anhui Provincial Key Laboratory of Hepatopancreatobiliary Surgery, Anhui Provincial Clinical Research Center for Hepatobiliary Diseases, Hefei, Anhui, China; School of Life Sciences, Division of Life Sciences and Medicine, University of Science and Technology of China, Hefei, Anhui 230001, China; Department of Hepatobiliary Surgery, State Key Laboratory of Immune Response and Immunotherapy, Centre for Leading Medicine and Advanced Technologies of IHM, The First Affiliated Hospital of USTC, Division of Life Sciences and Medicine, University of Science and Technology of China, Hefei, Anhui 230001, China; Anhui Provincial Key Laboratory of Hepatopancreatobiliary Surgery, Anhui Provincial Clinical Research Center for Hepatobiliary Diseases, Hefei, Anhui, China; School of Life Sciences, Division of Life Sciences and Medicine, University of Science and Technology of China, Hefei, Anhui 230001, China; Department of Oncological Surgery, Harbin Medical University Cancer Hospital, Harbin 150000, P.R. China; Department of Oncological Surgery, Harbin Medical University Cancer Hospital, Harbin 150000, P.R. China; Department of Hepatobiliary Surgery, State Key Laboratory of Immune Response and Immunotherapy, Centre for Leading Medicine and Advanced Technologies of IHM, The First Affiliated Hospital of USTC, Division of Life Sciences and Medicine, University of Science and Technology of China, Hefei, Anhui 230001, China; Anhui Provincial Key Laboratory of Hepatopancreatobiliary Surgery, Anhui Provincial Clinical Research Center for Hepatobiliary Diseases, Hefei, Anhui, China; School of Life Sciences, Division of Life Sciences and Medicine, University of Science and Technology of China, Hefei, Anhui 230001, China; Department of Hepatobiliary Surgery, State Key Laboratory of Immune Response and Immunotherapy, Centre for Leading Medicine and Advanced Technologies of IHM, The First Affiliated Hospital of USTC, Division of Life Sciences and Medicine, University of Science and Technology of China, Hefei, Anhui 230001, China; Anhui Provincial Key Laboratory of Hepatopancreatobiliary Surgery, Anhui Provincial Clinical Research Center for Hepatobiliary Diseases, Hefei, Anhui, China; School of Life Sciences, Division of Life Sciences and Medicine, University of Science and Technology of China, Hefei, Anhui 230001, China; Department of Hepatobiliary Surgery, State Key Laboratory of Immune Response and Immunotherapy, Centre for Leading Medicine and Advanced Technologies of IHM, The First Affiliated Hospital of USTC, Division of Life Sciences and Medicine, University of Science and Technology of China, Hefei, Anhui 230001, China; Anhui Provincial Key Laboratory of Hepatopancreatobiliary Surgery, Anhui Provincial Clinical Research Center for Hepatobiliary Diseases, Hefei, Anhui, China; School of Life Sciences, Division of Life Sciences and Medicine, University of Science and Technology of China, Hefei, Anhui 230001, China; Department of Hepatobiliary Surgery, State Key Laboratory of Immune Response and Immunotherapy, Centre for Leading Medicine and Advanced Technologies of IHM, The First Affiliated Hospital of USTC, Division of Life Sciences and Medicine, University of Science and Technology of China, Hefei, Anhui 230001, China; Anhui Provincial Key Laboratory of Hepatopancreatobiliary Surgery, Anhui Provincial Clinical Research Center for Hepatobiliary Diseases, Hefei, Anhui, China; School of Life Sciences, Division of Life Sciences and Medicine, University of Science and Technology of China, Hefei, Anhui 230001, China

## Abstract

G-quadruplex DNA (G4-DNA), a noncanonical tetrahelical nucleic acid structure stabilized by stacked G-quartets via Hoogsteen hydrogen bonding, plays critical roles in genomic regulation and disease pathogenesis. Current methodologies for detecting these structures face limitations in specificity, spatiotemporal resolution, and live-cell applicability. To address these challenges, we engineered G4-Flame, a genetically encoded fluorescent biosensor utilizing circularly permuted fluorescent protein technology. By strategically positioning a G4-specific binding domain proximal to the fluorophore of circularly permuted YFP (cpYFP), G4-Flame achieves real-time, high-resolution visualization of G4-DNA dynamics in living systems, with specificity across diverse G4 conformations. Experimental validation revealed distinct spatiotemporal patterns of G4-DNA during the cell cycle: nuclear G4-DNA levels peaked during the S phase, while mitochondrial G4-DNA was found to suppress the expression of mitochondrial-encoded genes. Clinically, serum analysis revealed significantly elevated G4-DNA levels in cancer patients compared to healthy controls. This work establishes G4-Flame as a transformative tool for investigating G4-DNA spatiotemporal regulation and advances its potential as a biomarker for early cancer detection, bridging fundamental research with clinical translation.

## Graphical Abstract

**Graphical Abstract:**
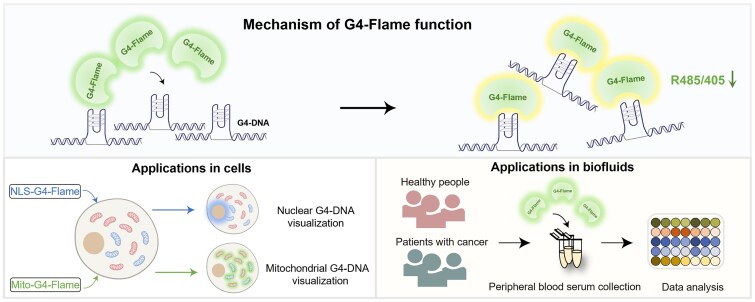

## Introduction

G-quadruplex DNA (G4-DNA) is a four-stranded helical structure formed by guanine-rich DNA sequences through Hoogsteen hydrogen bonds [1, 2], with its fundamental unit being the G-quartet, a planar arrangement of four guanine bases. This noncanonical nucleic acid structure exhibits significant polymorphism, including parallel, antiparallel, and hybrid conformations [3–5] (e.g. parallel propeller-type and antiparallel basket-type), whose stability is regulated by cations such as K^+^ and microenvironmental physicochemical properties [6–8]. At the genomic level, G4-DNA is specifically enriched in telomeres, oncogene promoter regions (e.g. c-MYC and BCL-2) [9–14], and recombination hotspots [15]. By regulating critical biological processes such as DNA replication, transcriptional elongation, and telomere homeostasis, G4-DNA is closely associated with the development of malignancies [16, 17], neurodegenerative diseases [18], and other major pathologies [19, 20].

As research into G4-DNA functions advances, elucidating its dynamic conformational transitions and pathological significance has become a forefront area of study. However, current detection technologies face significant limitations: traditional dyes like Thioflavin T (ThT) induce fluorescence enhancement upon G4 binding but suffer from poor specificity and high environmental sensitivity [21, 22]; DAOTA-M2 distinguishes G4 from other nucleic acid structures via fluorescence lifetime imaging microscopy (FLIM), yet its high equipment costs and operational complexity hinder scalable application [23, 24]; SiR-PyPDS, a fluorescent probe combining silicon rhodamine (SiR) with the G4-specific ligand pyridostatin (PDS), requires multistep organic synthesis with low yield (~30%) and challenging purification, limiting its large-scale use [25]. The most widely employed method, the G4-DNA-specific antibody BG4 [26], fails to meet the technical demands for real-time *in vivo* observation and suborganelle-level dynamic tracking. Additionally, although the nanobody SG4 [27] can enable visualization of G4-DNA in cells, its quantitative assessment of G4-DNA abundance is rather challenging. Thus, developing *in situ* detection tools with high spatiotemporal resolution is crucial for deeply elucidating the biological functions of G4-DNA.

Leveraging circularly permuted fluorescent proteins [28–32] molecular engineering, we developed G4-Flame (G4-DNA fluorescent labeled measurement effector), an ultrasensitive genetically encoded probe. By genetically reconfiguring circularly permuted YFP (cpYFP) to form a closed-loop structure through N- and C-terminal reconnection and introducing a G4-DNA-specific binding domain near the fluorophore, the resulting conformational rearrangement enables specific responses to G4-DNA structures. Experimental results demonstrate that G4-Flame not only specifically recognizes and responds to diverse G4-DNA conformations but also achieves high-resolution imaging in living cells. Using G4-Flame, we observed a significant increase in nuclear G4-DNA levels during the S phase, and the regulatory effects of mitochondrial G4-DNA changes on encoded gene expression were visualized. In clinical studies, serum G4-DNA levels in cancer patients were significantly higher than in healthy controls, highlighting its potential as a biomarker for early cancer screening. By integrating *in vivo* and *in vitro* detection systems, G4-Flame not only advances fundamental research into G4-DNA functions but also opens new avenues for translational medicine and clinical applications.

HEK293T, HCCLM3, and PLC cells were routinely cultured in DMEM (Proteintech, Cat #PM00031) supplemented with 10% fetal bovine serum and 1% penicillin–streptomycin (Solarbio, Cat #P1400). The cells were maintained in a humidified incubator at 37°C with 5% CO_2_. SNU 449 cells were cultured under the same conditions in RPMI-1640 medium (Proteintech, Cat #PM00032).

### Human subjects

The serum samples used in this study were obtained from patients at the First Affiliated Hospital of USTC (Hefei, Anhui, China). The collection process had no impact on the patients’ clinical diagnosis and treatment, nor did it cause any physical or mental burden to the patients. Since the clinical research protocol was approved by the Biomedical Ethics Committee of USTC (Approval No.: 2023-KY-191), the informed consent procedure was waived. This study fully complies with all relevant ethical guidelines, including the protection of patients’ personal information. It should be specifically noted that if a patient explicitly refuses to participate in this study, their samples or information will not be used.

### Transient transfection-based cell line construction

Three micrograms of plasmid DNA was taken and transfected into HEK293T cells in each well of a six-well plate using 4.5 μl of lipofection reagent (Yeasen, Cat #40802ES01), with a cell density of 50%~60% at the time of transfection. The medium was replaced 12 h post transfection for subsequent use, and cells were utilized within 2~5 days post transfection. Transiently transfected plasmids used are listed in Supplementary Table S1.

### Stable transfection-based cell line construction

PCDH lentiviral plasmids encoding the target protein were constructed. Lentiviruses were produced using a cotransfection strategy: HEK293T cells (with cell density controlled at 75%–90% at the time of transfection) were cotransfected with two lentiviral packaging vectors (pMD2.G and psPAX2), where the mass ratio of psPAX2:pMD2.G: target plasmid in the transfection system was 2:1:2; The volume-to-mass ratio of the liposome transfection reagent (Yeasen, Cat #40802ES01) to the total plasmid DNA was 1.5:1 (μl:µg). The supernatant containing viral particles was harvested 48 h post transfection. After 12 h of infection of the aforementioned cell lines (HEK293T, PLC, SNU 449, and HCCLM3), the virus-containing supernatant was removed, and cells were subsequently selected with 500 μg/ml G418 for one week or 5 µg/ml puromycin for 3 days. Stably transfected plasmids used are listed in Supplementary Table S1.

### Protein expression and purification

BL21(DE3) competent cells containing the plasmid expressing the target protein (G4-Flame) were cultured in LB media supplemented with 50 µg/ml streptomycin. An overnight culture was used to inoculate 1 liter of LB media containing 50 µg/ml streptomycin. Protein expression was induced by adding 1 mM Isopropyl β-D-1-thiogalactopyranoside (IPTG), and the culture was incubated at 16°C with shaking at 200 rpm overnight.

Bacterial cells were lysed using Tiechui lysis buffer (ACE Biotechnology, Cat #BR0005) on a rotator at 4°C for 20 min. After lysis, the lysates were centrifuged at 16 000 × *g* for 10 min at 4°C, and the supernatant was filtered through a 0.45-µm filter. The clarified lysate was then applied to a Ni-NTA sepharose column pre-equilibrated with binding buffer. The column was washed with five column volumes of binding buffer containing 10 mM imidazole (pH 8.0). The target protein was eluted with binding buffer supplemented with 200 mM imidazole (pH 8.0), and the buffer was exchanged to G4 buffer (10 mM Tris–HCL, 140 mM KCl, pH 7.4) by dialysis. Protein concentration was determined using an Amicon Ultra-15 Centrifugal Filter Unit with a 30-kDa cutoff (Millipore, Cat #UFC9010), and protein quantification was carried out using NanoDrop. Protein purity was assessed by sodium dodecyl sulfate–polyacrylamide gel electrophoresis (SDS–PAGE), and the samples were stored at −80°C until analysis. Plasmids used are listed in Supplementary Table S1.

### Absorption spectral scanning assay

An absorption spectral scanning experiment was performed on purified G4-Flame protein to detect characteristic absorption spectral changes upon its binding to G4-DNA. The procedure was as follows: 10 μM G4-DNA and 10 μM G4-Flame protein were mixed in G4 buffer (10 mM Tris–HCL, 140 mM KCl, pH 7.4). Absorption spectra were scanned over a wavelength range of 300–650 nm using a UV-Vis spectrophotometer (Hitachi, U-3900H), and the absorbance values at each wavelength were recorded. G4-DNA sequences used are listed in Supplementary Table S2.

### *In vitro* characterization of G4-DNA sensors

The purified G4-Flame protein was stored at −80°C until use. For *in vitro* assays, the sensor protein was diluted to final concentrations of 2 μM or 500 nM in 10 mM Tris–HCl buffer containing 140 mM KCl (pH7.5). Fluorescence detection was performed using a Synergy H1 multimode microplate reader (BioTek): emission spectra were recorded with 405 and 485 nm as excitation wavelengths, and the emission signal at 520 nm was simultaneously monitored.

For substrate titration, fluorescence intensity was measured using the same microplate reader equipped with a 405 nm excitation filter and a 485 nm emission bandpass filter. All reaction solutions were prepared in G4 buffer (10 mM Tris–HCL, 140 mM KCl, pH 7.4) immediately before use, and fluorescence signals were recorded without delay. DNA sequences used are listed in Supplementary Table S2.

### Electrophoretic mobility shift assay

Biotin-labeled oligonucleotides (Supplementary Table S2) were heated at 95°C for 5 min in 10 mM Tris–HCl containing 140 mM KCl (pH 7.5) and then cooled to 25°C at a rate of 1°C per minute. For G4-Flame, binding reactions were carried out in a 10 μl mixture containing 10 mM Tris–HCl, 140 mM KCl, and 5% glycerol (pH 7.4). At room temperature (RT), 10 nM biotin-labeled oligonucleotides were incubated with decreasing concentrations of the G4-Flame protein for 30 min. Samples were loaded onto an 8% nondenaturing polyacrylamide gel containing 70 mM KCl [acrylamide: bisacrylamide mass ratio 29:1 (wt/wt)] and electrophoresed in 0.5× Tris-Borate-EDTA buffer (TBE buffer) containing 200 mM KCl at 4°C, 100 V for 1.5 h. After electrophoresis, proteins were transferred to a positively charged nylon membrane by electrotransfer (wet transfer method) (transfer buffer: 0.5× TBE, 4°C, 100 V, 1.5 h), followed by UV crosslinking to fix the protein complexes. Finally, a chemiluminescent electrophoretic mobility shift assay (EMSA) detection kit (Beyotime; Cat #GS009) was used, following the manufacturer’s protocol, and imaging was performed using a chemiluminescence imaging system.

When using oligonucleotides simultaneously labeled with TAMRA or cy5.5 (Supplementary Table S2), the reaction samples can be directly analyzed using the LI-COR Odyssey dual-color infrared fluorescence imaging system after electrophoretic separation on an 8% nondenaturing polyacrylamide gel (acrylamide: bisacrylamide mass ratio 29:1).

### Circular dichroism

Circular dichroism (CD) spectroscopy was conducted using a Jasco J-1100 spectropolarimeter equipped with a thermoelectrically controlled cell holder. Oligonucleotides were dissolved in an annealing buffer to stabilize potential G4 structures. The samples were initially heated to 98°C and then slowly cooled to facilitate proper G4 folding. Subsequently, CD measurements were carried out in a quartz cell with an optical path length of 1 mm. Each CD spectroscopy measurement was the average of three scans collected within the wavelength range of 350–220 nm, with background correction applied to eliminate non-specific effects. The scan rate was set at 1 nm/step, and data were collected at an appropriate time interval to ensure accurate spectral acquisition.

### Isothermal titration calorimetry

The purified G4-Flame protein was diluted to a final concentration of 5 µM using a 10 mM Tris–HCl containing 140 mM KCl (pH 7.5). The corresponding oligonucleotides were dissolved in an annealing buffer to stabilize potential G4 structures and DNA duplexes. First, the samples were heated to 98°C and then slowly cooled to promote proper G4 folding and DNA duplex formation. After that, the oligonucleotides were diluted to 100 µM with the same G4 buffer for isothermal titration calorimetry (ITC) experiments, which were performed using a MicroCal PEAQ-ITC instrument. The experiments were carried out at 25°C. Each titration involved a series of 2-μl injections of the reactant into the sample cell containing the G4-Flame solution, with an interval of 120 s between consecutive injections. The raw data were analyzed using Origin software to calculate the binding constant (*Kd*​), binding enthalpy (Δ*H*), and entropy change (Δ*S*).

### Nuclear magnetic resonance spectroscopy

One-dimensional proton nuclear magnetic resonance (^1^H NMR) spectra of 0.2 mM DNA samples (purchased from Generalbiol) were recorded at 298 K using a 500 MHz Bruker spectrometer equipped with a cryoprobe. The DNA samples were prepared in a 20 mM potassium phosphate buffer containing 100 mM KCl, adjusted to pH 6.8.

To promote the formation of G-quadruplex structures, the samples were subjected to heat treatment by incubation at 95°C for 5 min, followed by slow cooling to RT. Before nuclear magnetic resonance (NMR) analysis, 10% deuterium oxide (D_2_O) was added to the samples for field-frequency locking. All NMR datasets were processed and analyzed using Bruker Topspin 3.6.2 software.

### Microscale thermophoresis

G4-Flame probe with an initial concentration of 20 μM was taken, and a two-fold serial dilution (two-fold dilution each time) was performed to prepare 12 gradient concentration samples. The diluted G4-Flame probe was mixed with Cy5.5-labeled G4-DNA at a 1:1 volume ratio, ensuring that the final concentration of G4-DNA in the mixture was 20 nM and the maximum final concentration of the G4-Flame probe was 10 μM. After mixing, capillary loading was performed, and detection was carried out using a Monolith NT.115 instrument.

### CUT&tag

Briefly, cells were harvested by centrifugation at 900 × *g* for 3 min at RT. A total of 1 × 10^5^ cells were resuspended in 150 μl of wash buffer, which consisted of 20 mM HEPES, 150 mM NaCl, and 0.5 mM spermidine. Then, the cells were incubated with activated ConA Beads (Vazyme, Cat #N515) for 10 min at RT to promote binding.

Next, the cells were incubated overnight at 4°C with a prediluted primary antibody solution. After two rapid washes with the wash buffer, the cells were incubated with a secondary antibody dilution for 1 h at RT. Following another two quick washes, the cells were incubated with pAG-Tn5 transposase (Vazyme, Cat #S614) for 1 h at RT.

The samples were then washed twice with 300-wash buffer (containing 20 mM HEPES, 300 mM NaCl, and 0.5 mM spermidine). For tagmentation, the samples were resuspended in 100 μl of tagmentation buffer (300-wash buffer supplemented with 1 mM MgCl_2_) and reacted at 37°C for 1 h. The fragmentation reaction was stopped by adding 0.5 M ethylenediaminetetraacetic acid, 10% SDS, and proteinase K (Sangon, Cat #B600169). DNA extraction was then performed using phenol reagent (Solarbio, Cat #T0250).

The extracted DNA was amplified using custom-designed primers and 2× HiFi AmpliMix (Novoprotein, Cat #E089). Polymerase chain reaction (PCR) products were purified with DNA clean beads (Vazyme, Cat #N411). Library quality control and sequencing were carried out by Waker Bioscience and Novogene.

For data analysis, following the previously described methodology [33], adapter trimming and removal of low-quality bases from paired-end reads were conducted using Trim Galore software. Clean reads were then aligned to the hg38 reference genome using Bowtie2, and PCR duplicates were removed with Picard. Genome browser tracks in bigWig format were generated from merged replicates using samtools, and peak calling was performed with MACS2 using the parameters -f BAMPE-broad-nomodel. The resulting bigWig data were visualized using the Integrative Genomics Viewer software from the Broad Institute, while peak visualizations were prepared using Deeptools.

The sequencing data have been deposited in the National Center for Biotechnology Information (NCBI) Gene Expression Omnibus (GEO) database under the accession number GSE314730.

### Immunofluorescence

Cells expressing the nuclear localization signal (NLS)–G4-Flame sensor or Mito-G4-Flame were seeded in confocal dishes. After 24 h of culture, the cells were washed twice with phosphate-buffered saline (PBS) buffer and fixed with 4% paraformaldehyde for 20 min at RT. Subsequently, the cells were permeabilized with 0.2% triton X-100 and blocked with 5% bovine serum albumin (BSA).

Next, cells were stained with G4-DNA-specific antibody BG4, mitochondrial probe ATP5A1, mito-tracker, DHX36, or PI as required for specific experimental purposes, followed by fluorescence imaging. For cells without special staining requirements, fluorescence imaging of G4-Flame was performed after fixation. Finally, images were acquired using a Leica STED confocal microscope.

### RNA-seq

Total RNA was extracted from cells, and libraries were prepared using the VAHTS Universal V6 RNA-seq Library Prep Kit for Illumina^®^ following the standard Illumina instructions. Before sequencing on an Illumina NovaSeq 6000, the concentration and size distribution of the complementary DNA (cDNA) libraries were assessed using the Agilent 4200 Bioanalyzer in accordance with the manufacturer’s protocols.

Raw reads were quality-filtered using Seqtk and then aligned to the reference genome with Hisat2 (version 2.0.4). Gene fragment counts were generated using StringTie (v1.3.3b) and normalized by the trimmed mean of M-values (TMM). Differential expression analysis was performed using edgeR. Significant differentially expressed genes were identified as those with a false discovery rate value below the threshold (Q < 0.05) and a fold-change greater than 2. The sequencing data have been deposited in the NCBI GEO database under the accession number GSE314730. The services were commercially provided by Shanghai Biotec Service Co., Ltd.

### Western blot

Purified G4-Flame samples were subjected to SDS–PAGE and then transferred to nitrocellulose membranes. The membranes were blocked with 5% defatted milk for 1 h at RT and subsequently incubated overnight at 4°C with the indicated primary antibodies (Supplementary Table S3). After washing, the membranes were incubated with species-matched horseradish peroxidase (HRP)-coupled secondary antibodies at RT for 40 min. Protein bands were visualized using enhanced chemiluminescence reagent (SUDGEN) and recorded using a chemiluminescence imaging system (Tanon).

### ATP assay

The HEK293T cell line was transiently transfected with the pcDNA-Mito-G4-Flame plasmid. After a 24-h transfection period, the cells were lysed, and the ATP concentration in the cell lysates was measured using the Beyotime Enhanced ATP Assay Kit. To ensure the accuracy of the data, the ATP content of all samples was normalized to the total protein content, which was determined using the bicinchoninic acid protein quantification method. This normalization step accounted for potential variations in cell number among different samples.

### Mitochondrial membrane potential assay

To evaluate the intracellular mitochondrial membrane potential, the HEK293T cell line was transiently transfected with the pcDNA-Mito–G4-Flame plasmid. Twenty-four hours after transfection, the mitochondrial membrane potential was measured using the Beyotime Enhanced Mitochondrial Membrane Potential Assay Kit with JC-1.

### G4-Flame-based serum G4-DNA analysis

For sensor-based G4-DNA analysis, all assays were performed in 384-well black-bottom plates. Five microliters of sample and 5 μl of G4-Flame protein (final concentration: 500 nM) were sequentially added to each well. Fluorescence intensity was immediately measured using a Synergy H1 multimode microplate reader (BioTek) with excitation wavelengths set at 405 and 485 nm, and an emission wavelength of 520 nm (band-pass filter). Background fluorescence was determined by measuring the fluorescence of a solution prepared by mixing BSA and serum samples at a 1:1 ratio, both at the same final concentration. Data are presented as either a representative result from at least three repeated experiments or the mean value from three or more independent experiments. Patient information is listed in Supplementary Table S4.

### Colony formation

The indicated cells were first seeded in triplicate into six-well plates at a density of 1000 cells per well. After 10 days of culture, the resulting colonies were fixed with 4% paraformaldehyde for 10 min at RT, followed by staining with Crystal Violet Staining Solution (0.5% w/v crystal violet, Sigma–Aldrich; 25% methanol) for 20 min at RT. The wells were washed with deionized water (ddH_2_O) and allowed to dry overnight. Subsequently, 1 ml of 10% acetic acid (Sinopharm) was added to each well and incubated at RT for 1 h to extract the stain. Each sample (100 μl) was then transferred to a 96-well plate, and the absorbance at 600 nm was measured using a Synergy H1 microplate reader (BioTek).

### Quantitative real-time PCR

Total RNA was extracted using an RNA extraction kit (TIANGEN, Cat #DP419) following the manufacturer’s protocol and reverse-transcribed into cDNA using a reverse transcription kit (Takara, Cat #RR036A). Quantitative real-time PCR (qPCR) was performed on an Agilent Mx300/5P system (Roche) using SYBR Master Mix. Each 20 μl reaction contained 5 μl cDNA, 1 μl primers, 10 μl SYBR Master Mix (YEASEN, Cat #11185ES03), and 4 μl double-distilled water (ddH_2_O). Gene expression levels were normalized to the internal reference gene actin (actin) and calculated using the 2^−ΔΔCt^ method. All reactions were performed in triplicate. Primer sequences used are listed in Supplementary Table S2.

### Detection of cell viability

Cell viability and proliferation were measured using the Cell Counting Kit-8 (Bimake, Cat #B34302) according to the manufacturer’s instructions. Briefly, 1000 cells per well were seeded in triplicate into 96-well plates and allowed to adhere overnight. Cell viability was assessed daily for six consecutive days starting from the next day after seeding.

### Cell cycle analysis by PI staining​

Cells were seeded in 6-cm culture dishes and allowed to adhere overnight. For cell cycle synchronization, cells were treated with thymidine (2 mM) to arrest the cell cycle at the G1/S boundary. To obtain S-phase-enriched cells, cells were collected 2 h after thymidine treatment. To obtain G1-phase-enriched cells, thymidine was washed out with PBS, and cells were released into fresh complete medium and allowed to progress into G1 phase for the indicated time before harvesting. Subsequently, cells were harvested and washed once with PBS. Cell pellets were resuspended in 300 μl PBS and fixed by dropwise addition of 700 μl ice-cold anhydrous ethanol, followed by overnight incubation at 4°C. The next day, cells were centrifuged at 300 × *g* for 6 min, and the supernatant was discarded. Cells were permeabilized in 500 μl 0.5% triton X-100 for 30 min at RT. Subsequently, cells were stained with 20 µg/ml propidium iodide (PI) and 0.1 mg/ml RNase A for 15 min at RT in the dark. After centrifugation, pellets were resuspended in 300 μl PBS and analyzed on a Beckman Coulter CytoFLEX flow cytometer.

## Results

### Engineering of G-quadruplex sensors G4-Flame

**Figure 1.**
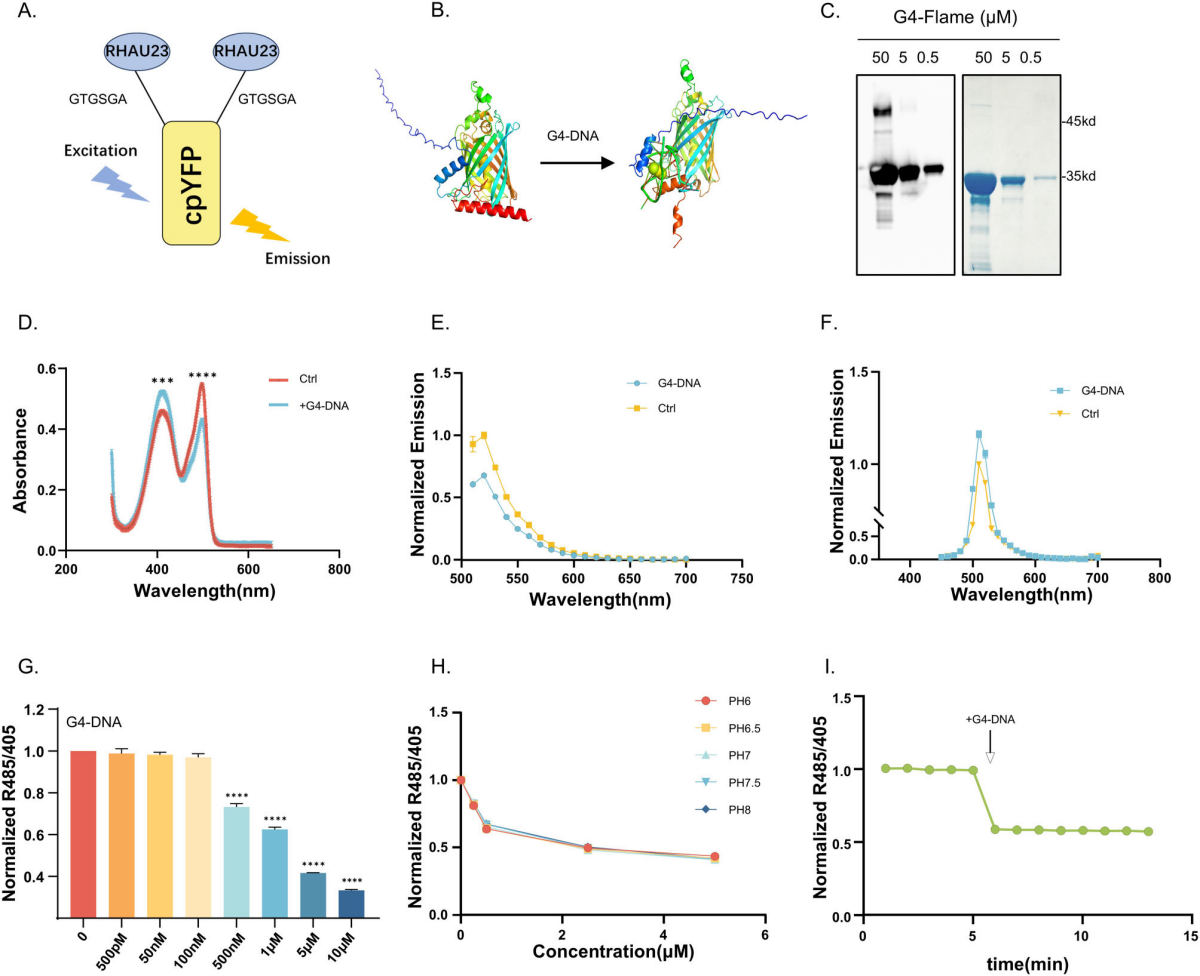
Engineering of G-quadruplex sensors. (**A, B**). Schematic representation of the G4-DNA sensor G4-Flame. The fluorescent protein component of G4-Flame was inserted at both the N- and C-termini of the G4-DNA binding protein domain RHAU23. AlphaFold3 predicts structural changes in G4-Flame upon G4-DNA binding. (**C**) Western blot (WB) analysis confirmed G4-Flame protein purification, with Coomassie staining demonstrating high purity of the preparation. (**D**) Absorbance spectra of the G4-Flame (10 μM) in the absence and presence of G4-DNA (10μM) (*n* = 3). (**E, F**) Fluorescence intensities of G4-Flame with excitation wavelengths of 485 or 405 nm in the presence of G4-DNA (*n* = 3). (**G**) Fluorescence response (R485/405 ratio) of G4-Flame at the indicated concentration of G4-DNA (probe concentration: 500 nM). Data are normalized to the initial value (*n* = 3). (**H**) Fluorescence response (R485/405 ratio) of G4-Flame as a function of G4-DNA concentration at specified pH values (probe concentration: 500 nM). Data are normalized to the initial value (*n* = 3). (**I**) Kinetics of fluorescence response of G4-Flame (R485/405 ratio) upon addition of G4-DNA (*n *= 3). Data information: (D–I) are mean ± SD, (D) unpaired *t-*test, (G) one-way ANOVA with Dunnett’s multiple comparisons test; ns, not significant, *P < .05, **P < .01, ***P < .001, ****P < .0001.

G4-DNA is a noncanonical nucleic acid structure formed by stacked guanine tetrads stabilized by Hoogsteen hydrogen bonding and monovalent cations. G4-DNA can form in guanine-rich regions of the genome and is implicated in regulating key biological processes such as transcription, replication, genome stability, and telomere maintenance. Given the critical regulatory roles of G4-DNA in these fundamental processes, developing genetically encoded sensors capable of specifically recognizing and reporting its presence and subcellular localization in living cells has emerged as a priority for dissecting its dynamic functions. The design of G4-Flame is based on the cpYFP fluorescent protein scaffold, constructed by fusing the RHAU23 G4-DNA recognition domain [34–36] to its N- and C-termini (Fig. 1A). The RHAU23 domain corresponds to amino acids 53–75 of the DHX36 protein, which not only specifically binds to G4-DNA but has also been widely applied in existing studies related to G4-DNA. AlphaFold3 structure prediction revealed that this chimeric protein adopts a clamp-like conformation for specific recognition of G4-DNA (Fig. 1B). Purified G4-Flame protein (Fig. 1C) exhibited characteristic absorption spectral changes upon binding to G4-DNA: a significant reduction in the absorbance peak at 485 nm, accompanied by an enhancement of absorption around 405 nm (Fig. 1D). Parallel fluorescence spectral scanning confirmed these observations (Fig. 1E and F). Normalization of the data yielded an R485/405 ratio that decreased monotonically with increasing G4-DNA concentration (Fig. 1G and Supplementary Fig. S1A), providing a robust molecular basis for sensitive and quantitative G4-DNA detection.

Further, specificity assays via gradient dilution established a standard curve (Supplementary Fig. S1B) demonstrating nanomolar (nM) detection sensitivity. pH-dependent analysis using a gradient buffer system (Fig. 1H) revealed that the normalized G4-Flame response remained stable across the physiological pH range (6–8), indicating robust performance unaffected by physiological pH fluctuations. Kinetic studies (Fig. 1I) further showed rapid response (<60 s) to G4-DNA concentration changes with signal stability maintained for >10 min. Together, these properties support the use of G4-Flame as a reliable molecular probe for real-time monitoring of G4-DNA dynamics in biological systems.

### Specific recognition and binding by G4-Flame enables it to respond fluorescently to G4-DNA

**Figure 2.**
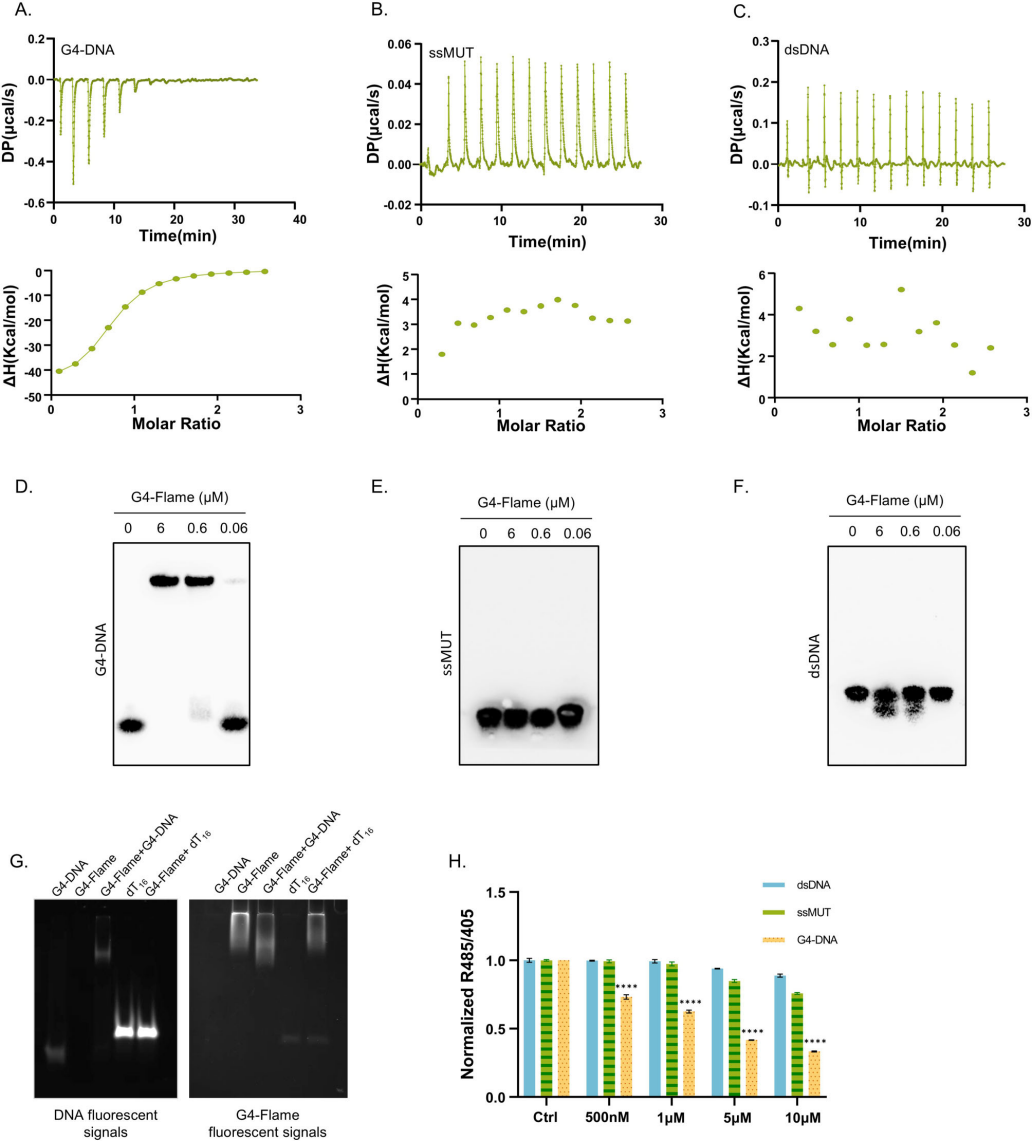
G4-Flame specifically binds and responds to G4-DNA structures. ITC analysis of G4-Flame binding to different DNA topologies: (**A**) G4-DNA, (**B**) ssMUT, and (**C**) dsDNA. Upper panels show the raw ITC thermograms obtained by titrating each DNA into purified G4-Flame in G4 buffer, and lower panels show the corresponding integrated binding isotherms plotted as a function of molar ratio. The data were fitted using a one-site binding model to determine the binding enthalpy (Δ*H*). A clear binding signal was observed for G4-DNA, whereas ssMUT and dsDNA showed no detectable binding. EMSA analysis of G4-Flame binding specificity using 10 nM biotinylated DNA substrates: (**D**) G4-DNA, (**E**) ssMUT, and (**F**) dsDNA. Protein–DNA complexes were resolved by native PAGE and detected via streptavidin–HRP chemiluminescence. A pronounced mobility shift was observed for G4-DNA upon incubation with G4-Flame, whereas ssMUT and dsDNA showed no detectable complex formation. (**G**) Binding specificity of G4-Flame assessed by fluorescence shift assay. Two micromolar TAMRA-labeled G4 DNA showed interaction with 2 µM G4-Flame after 5 min incubation at RT. No detectable interaction was observed between 2 µM G4-Flame and 10 µM TAMRA-labeled oligo dT16 single-stranded DNA. Fluorescent signals were captured using a gel documentation system. (**H**) Fluorescence response (R485/405 ratio) of G4-Flame at the indicated concentrations of G4-DNA, ssMUT, and dsDNA (*n* = 3). The R485/405 ratio decreased progressively with increasing concentrations of G4-DNA, whereas no comparable change was observed for ssMUT or dsDNA. Data information: (H) are mean ± SD, one-way ANOVA with Dunnett’s multiple comparisons test; ns, not significant; *P < .05, **P < .01, ***P < .001, ****P < .0001.

To systematically evaluate the recognition specificity of G4-Flame toward G4-DNA, we employed multidimensional experimental approaches to evaluate its binding selectivity. ITC analysis demonstrated that G4-Flame binds to G4-DNA with a characteristic saturation isotherm, yielding a dissociation constant (*K*_d_) of 449 nM (Fig. 2A), indicative of hundreds nanomolar-level affinity. Simultaneously, microscale thermophoresis experiments also demonstrated that G4-Flame binds to parallel G4-DNA with an affinity in the hundred-nanomolar range (Supplementary Fig.  S1I). No significant binding was observed upon incubation with single-stranded mutant DNA (ssMUT) or double-stranded DNA (dsDNA) (Fig. 2B and C). EMSA revealed specific binding bands exclusively in the presence of both G4-Flame and G4-DNA (Fig. 2D), where the binding efficiency exhibited a linear decline with decreasing protein concentration. The G4-Flame–G4-DNA complex migrated to a higher position compared to free G4-DNA, providing direct evidence for their interaction. In contrast, neither ssMUT nor dsDNA exhibited band shifts (Fig. 2E and F), confirming the absence of interaction with G4-Flame. Similarly, single-stranded oligo dT and double-stranded Dickerson DNA (Fig. 2G and Supplementary Fig. S1G) showed no mobility shifts, further excluding nonspecific binding to these nucleic acids. To strictly confirm G4-Flame’s selectivity toward G4-DNA, competition assay was applied as follows. Cyc5.5-labeled ssMUT and dsDNA together with equal amount of TAMRA-labeled G4-DNA were mixed in one tube simultaneously. The mixture wase titrated with G4-Flame then. Only G4-DNA was preyed among the DNA mixture (Supplementary Fig. S1H). It perfectly demonstrated that the interaction between G4-Flame and G4-DNA was structurally dependent. ssMUT/dsDNA/G4-DNA were also tested by titration with G4-Flame separately. Also, only G4-DNA displayed obvious binding of G4-Flame while neither of ssMUT or dsDNA did not bind G4-Flame at all.

Fluorescence spectroscopy further demonstrated that the probe exhibited a pronounced response upon binding to G4-DNA at a concentration of 500 nM (Fig. 2H), with a response intensity far exceeding that observed for ssMUT or dsDNA. In contrast, dual-channel fluorescence signals (485/405 nm) showed no significant changes for ssMUT or dsDNA at low concentrations (Supplementary Fig. S1C–F), with only a slight response detected at 5 μM. Collectively, these results establish the specific recognition of G4-DNA structures by G4-Flame.

### G4-Flame recognizes different topological forms of G4-DNA structures

**Figure 3.**
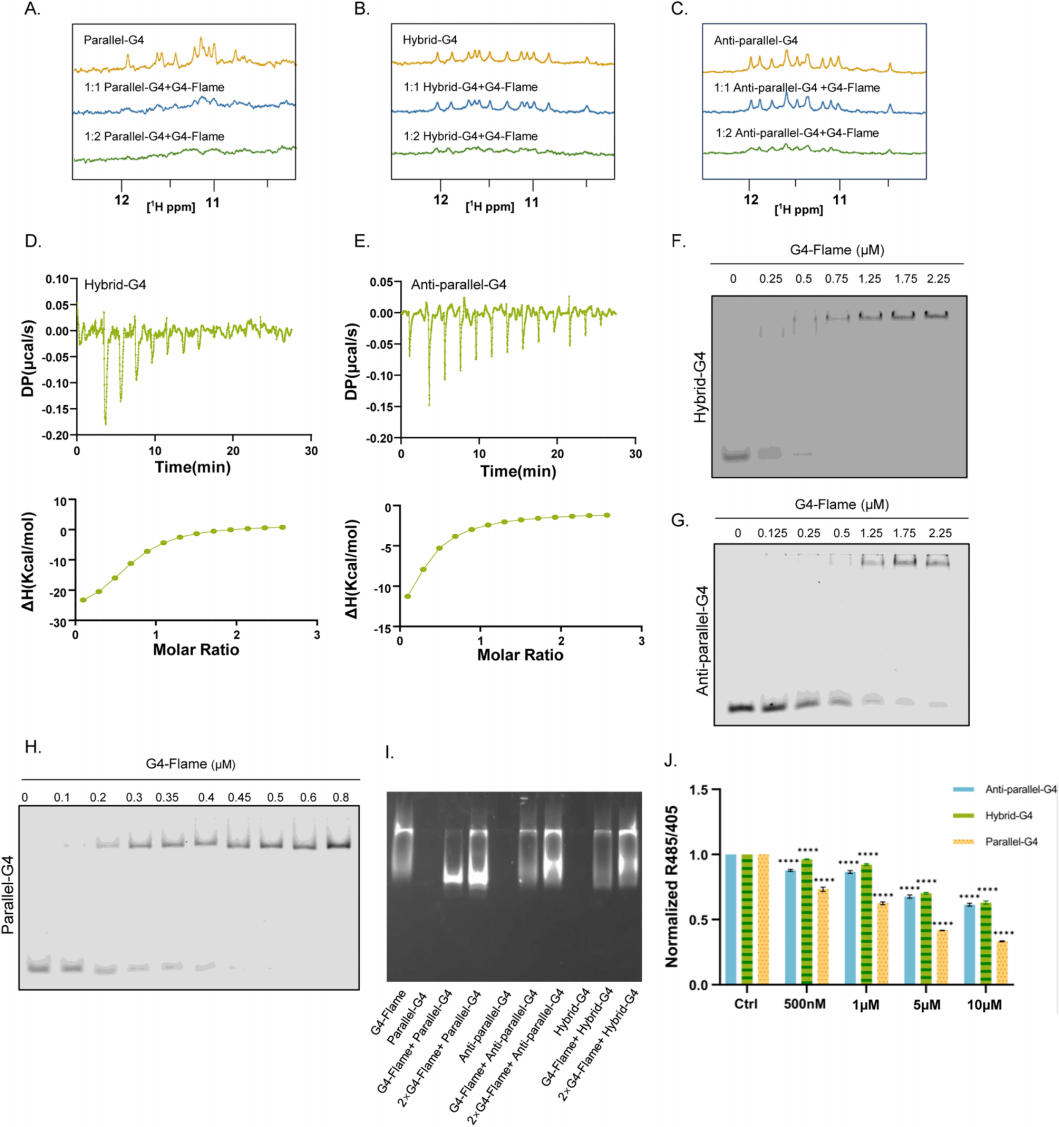
G4-Flame exhibits specific binding activity toward multiple G4-DNA topologies. NMR spectroscopy was used to characterize G4-Flame’s interactions with different G4-DNA topologies: (**A**) parallel, (**B**) hybrid, and (**C**) antiparallel structures. Upon incremental addition of G4-Flame, the imino proton signals of all three G4-DNA conformations progressively decreased in intensity. ITC analysis of G4-Flame binding to different G4-DNA topologies: (**D**) hybrid and (**E**) antiparallel structures. Upper panels show the raw ITC thermograms obtained by titrating each G4-DNA into purified G4-Flame in G4 buffer, and lower panels show the corresponding integrated binding isotherms plotted as a function of molar ratio. The data were fitted using a one-site binding model to determine the binding enthalpy (Δ*H*). Clear binding signals were observed for both hybrid and antiparallel G4-DNA structures. EMSA analysis of G4-Flame binding specificity using 500 nM TAMRA-labeled DNA substrates: (**F**) hybrid, (**G**) antiparallel, and (**H**) parallel structures. The complexes were resolved by native PAGE and visualized using a fluorescence imaging system. A pronounced mobility shift was observed for all tested G4-DNA topologies upon incubation with G4-Flame. (**I**) Native PAGE analysis of G4-Flame–G4 interactions. Two micromolar G4-DNA (unlabeled) were incubated with 2 or 4 µM G4-Flame at RT for 5 min. The resulting samples were applied to native PAGE. Four micromolar of Flame were loaded as control. Signal from fluorescent of Flame was monitored. Upon incubation with G4-DNA, a mobility shift of G4-Flame was observed. (**J**) Fluorescence response (R485/405 ratio) of G4-Flame at the indicated concentrations of parallel-G4, hybrid-G4, and antiparallel-G4 (*n* = 3). The R485/405 ratio decreased progressively with increasing concentrations of G4-DNA, with the largest decrease observed for parallel G4-DNA. Data information: (J) are mean ± SD, two-way ANOVA with Dunnett’s multiple comparisons test; ns, not significant; *P < .05, **P < .01, ***P < .001, ****P < .0001.

To comprehensively evaluate the recognition ability of G4-Flame for different topological G4-DNA structures, this study investigated its responsiveness to each conformation through multimodal experiments. NMR [36–38] titration experiments revealed characteristic chemical shift perturbations upon addition of G4-Flame to parallel, hybrid, and antiparallel G4-DNA conformations (Fig. 3A–C). Among the observed signals, the guanine imino proton signal exhibited the most pronounced changes in the presence of parallel G4-DNA, indicating a relatively strong interaction with this conformation, while notable perturbations were also detected for the hybrid and antiparallel forms, demonstrating that G4-Flame is capable of recognizing all three topologies.

ITC was performed. The measured *K*_d_ showed that G4-Flame bound to parallel G4-DNA with the highest affinity (*K*_d_ = 449 nM), and also formed stable complexes with hybrid (*K*_d_ = 759 nM) and antiparallel (*K*_d_ = 2.5 μM) forms (Figs 2A and 3D and E), in agreement with the NMR data. These values reflect a gradual variation in binding strength rather than an exclusive preference and confirm that G4-Flame can engage each conformation effectively. EMSA further supported this conclusion: all three G4-DNA conformations displayed concentration-dependent gel retardation in the presence of G4-Flame (Fig. 3F–H), with the degree of retardation following the order parallel > hybrid > antiparallel. Native polyacrylamide gel electrophoresis (Native PAGE) yielded consistent results (Fig. 3I), again indicating that G4-Flame interacts detectably with each topology.

In addition to affinity measurements, fluorescence spectroscopy was used to evaluate the functional response of G4-Flame. Under dual-excitation wavelengths (485/405 nm), G4-Flame produced a clear ratiometric fluorescence response not only with parallel G4-DNA but also with hybrid and antiparallel forms (Supplementary Fig.  S2A–H). The most pronounced ratiometric shift occurred with the parallel conformation, correlating with its relatively higher binding affinity (Fig. 3J). Together, these results establish that G4-Flame possesses recognition capability for parallel, hybrid, and antiparallel G4-DNA structures, with binding affinities that vary in a continuous manner among the three conformations.

### G4-Flame probe variants and their fluorescence responses to different DNA types

To develop G4-DNA probes with higher sensitivity and specificity, we designed a series of variants (including G4-Flame): by inserting either one or two intact RHAU23 proteins at both ends of the yellow circular fluorescent protein, or by inserting two truncated subunits, and systematically naming the resulting constructs (Supplementary Fig. S3A). Subsequently, for each variant, the absorption spectrum changes upon addition of G4-DNA were measured. The results showed that all six G4-Flame probe variants exhibited characteristic absorption peaks around 485 and 405 nm (Supplementary Fig. S3B–G); among them, G4-Flame-1, G4-Flame-2, G4-Flame-3, and G4-Flame-N displayed an increase in the peak at 405 nm after G4-DNA addition, while G4-Flame-2, G4-Flame-4, and G4-Flame-C showed a decrease in the peak at 485 nm after G4-DNA addition (Supplementary Fig. S3B–G).

Furthermore, for each variant, the changes in fluorescence intensity ratio (R485/405) upon addition of different types of DNA (including non-G4-structured dsDNA and ssMUT DNA) and various types of G4-DNA were measured. The experimental results indicated that G4-Flame-1, G4-Flame-2, G4-Flame-3, and G4-Flame-4 exhibited a relatively low response to all three types of DNA (non-G4 dsDNA, ssMUT DNA, and G4-DNA); however, when cpYFP was linked to only a single RHAU23 domain (G4-Flame-N and G4-Flame-C), their response to ssMUT and dsDNA was significantly reduced, and they exhibited specific recognition for parallel-type G4-DNA—this characteristic makes them ideal foundational probes for subsequent studies (such as specificity optimization or mechanistic analysis) (Supplementary Fig. S3H and I).

### G4-Flame exhibits biocompatibility and minimal cellular interference

To systematically assess the biocompatibility of the G4-Flame, this study conducted CCK8 proliferation assays and colony formation experiments. No significant differences were observed in colony-forming abilities (Supplementary Fig. S4A and B) or proliferation rates (Supplementary Fig. S4C) between cells expressing G4-Flame and control groups, indicating that the probe does not interfere with cell growth. Deep transcriptome sequencing analysis (Supplementary Fig. S4D and E) further revealed that overexpression of G4-Flame induced significant changes in only 0.3% of genes (|log_2_FC|>1, *P *< 0.05), confirming its low-interference properties. CD spectroscopy analysis showed that, before and after addition of G4-Flame, the characteristic CD signals of the three major G4-DNA [37] topologies—parallel, hybrid, and antiparallel—remained unchanged: the positive peaks at ~260 and 295 nm and the negative peak at 240 nm exhibited no shift or change in shape (Supplementary Fig. S4F–H), with only a slight increase in signal intensity. These results indicate that binding of G4-Flame does not induce structural transitions in G4-DNA and does not compromise its integrity. Importantly, CD melting (CD-Tm) experiments showed that the presence of G4-Flame does not significantly change the melting temperature of G4-DNA, indicating that G4-Flame does not measurably affect the thermodynamic stability of folded G4 structures (Supplementary Fig. S4I). This unique nondisruptive binding mode, combined with its hundreds nanomolar-level affinity, allows G4-Flame to maintain the native state of the target structure while enabling highly sensitive detection during dynamic monitoring.

### Application of nuclear-localized G4-Flame for monitoring G4-DNA dynamics in living cells

**Figure 4.**
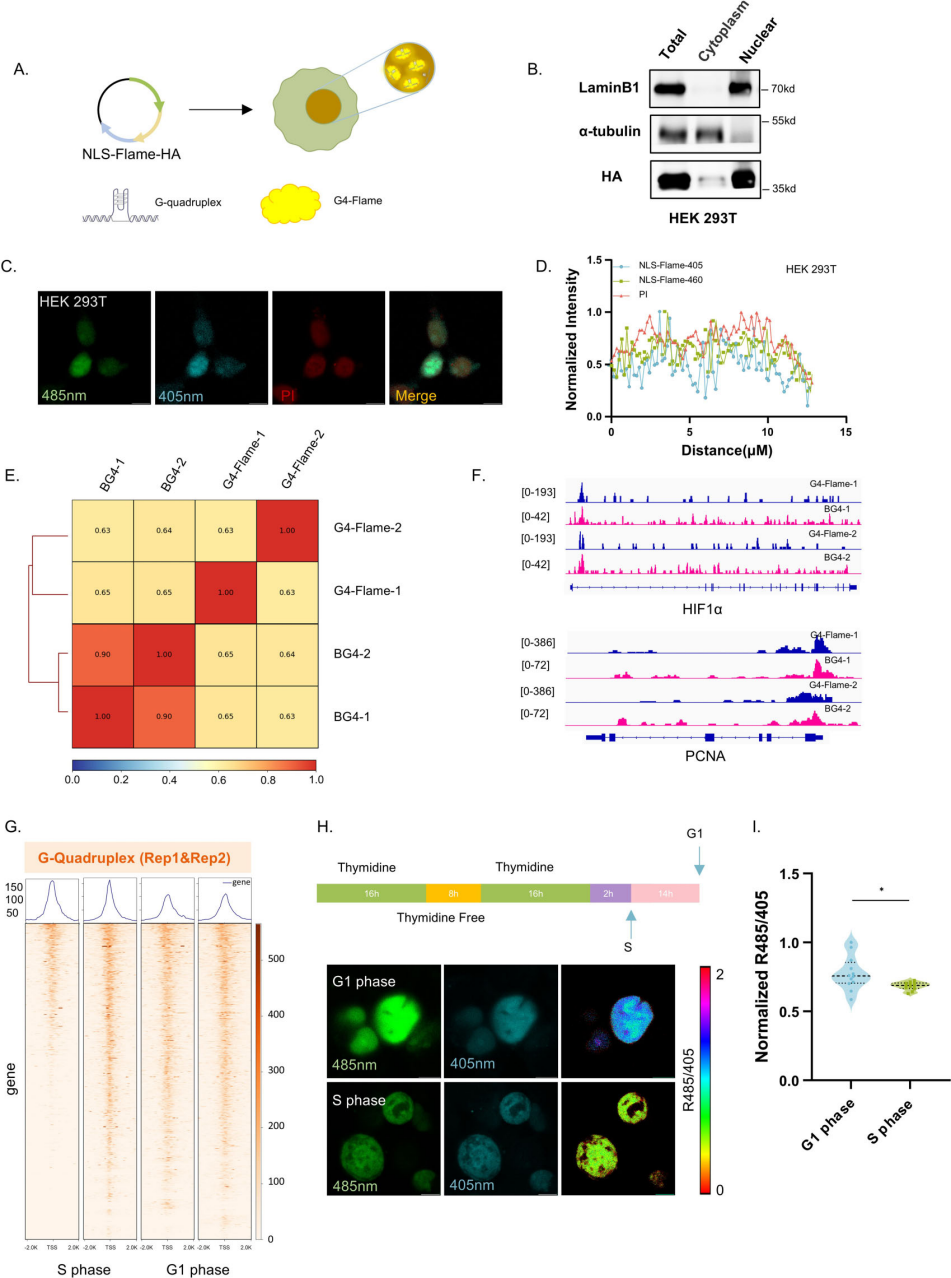
G4-Flame visualizes G4 Structures in cell nuclei. (**A**) Schematic Diagram of NLS–G4-Flame cell Line construction. Green:NLS sequence, yellow:G4-Flame sequence, and blue:HA tag sequences. Arrows of different colors in the plasmid represent the corresponding functional elements shown in the diagram. (**B**) Subcellular fractionation assay validating NLS–G4-Flame nuclear targeting. WB probed with: anti-Lamin B1 (nuclear marker), anti-α-tubulin (cytoplasmic marker), and anti-HA (detecting G4-Flame). Results confirm exclusive nuclear localization of HA–G4-Flame in stably transfected HEK293T cells. Fluorescence images (**C**) and co-localization analysis (**D**) of 293T cells transiently expressing NLS-G4-Flame (ex 485/405 nm, em 520 nm) with PI nuclear counterstain, showing clear nuclear localization. Scale bars: 10 µm. (**E**) Genome-wide consistency analysis of G4-DNA recognition by G4-Flame and BG4 in SNU449 cells using CUT&Tag. G4-Flame occupancy (anti-HA) was compared with endogenous G4 detection (BG4 antibody), and genome-wide correlation was assessed using Spearman correlation. (**F**) Genome browser visualization of G4-Flame (anti-HA) and endogenous G4-DNA (BG4 antibody) occupancy profiles across the target genomic region (HLF1α, PCNA). (**G**) Genome-wide G4-DNA occupancy around transcription start sites (±2 kb) in SNU449 cells, analyzed by CUT&Tag, comparing cells arrested in G1 versus S phase. Overall G4-DNA abundance was lower in G1-phase cells than in S-phase cells. (**H, I**) Cell cycle-dependent changes in nuclear G4-DNA in HEK293T cells transiently expressing NLS–G4-Flame. Cells were synchronized in G1 and S phase using a thymidine double-block protocol. Fluorescence images show nuclear G4-DNA dynamics, with quantitative analysis of R485/405 ratios indicating a decrease in S-phase cells. Scale bars: 10 µm. Data information: (**I**) unpaired *t*-test, *P < .05.

To systematically elucidate the dynamic regulatory mechanisms of G4-DNA within mammalian cell nuclei, we engineered an NLS–G4-Flame sensor by integrating an NLS (Fig. 4A). Nuclear-cytoplasmic fractionation assays and immunofluorescence analyses confirmed the specific nuclear localization of NLS–G4-Flame in HEK293T cells (Fig. 4B–D), with consistent nuclear localization patterns observed in hepatocellular carcinoma cell lines SNU-449 and PLC (Supplementary Fig. S5A and B). Leveraging the HA tag incorporated into nuclear-localized G4-Flame, we performed CUT&Tag assays using an anti-HA antibody to map its genome-wide binding landscape. The resulting binding profile showed consistent results with the genomic occupancy pattern of the G4-DNA-specific antibody BG4 (Fig. 4E and F), thereby validating both the specific recognition capability of G4-Flame toward G-quadruplexes and its reliability for live-cell detection. Co-localization imaging of NLS–GF-Flame and the widely used G4-DNA antibody BG4 revealed partial spatial exclusion within the nucleus, suggesting that the two probes may compete for binding to overlapping G4-DNA epitopes. This phenomenon was consistently observed in both PLC and SNU 449 cell lines (Supplementary Fig. S5D and E).

It has been previously reported that G4-DNA levels increase during S phase [25–32,34–38]. Consistent with this, our cell cycle synchronization experiments confirmed that G4-DNA levels were significantly elevated in S phase compared to G1-phase cells (Fig. 4G), a finding verified by BG4 fluorescence staining (Supplementary Fig. S5C). This dynamic variation was directly captured using the transiently expressed NLS–G4-Flame sensor​ (Fig. 4H and I), supporting the notion that G4-DNA is actively regulated during DNA replication. In addition, flow cytometry analysis confirmed the effectiveness of cell cycle synchronization (Supplementary Fig. S5F and G). Collectively, these findings demonstrate that the NLS–G4-Flame sensor enables *in situ* real-time monitoring of G4-DNA dynamics within living cells, while providing an innovative visualization tool for investigating the biological functions of G4-DNA.

### Mito-G4-Flame reveals mitochondrial G4-DNA suppresses mtDNA expression

**Figure 5.**
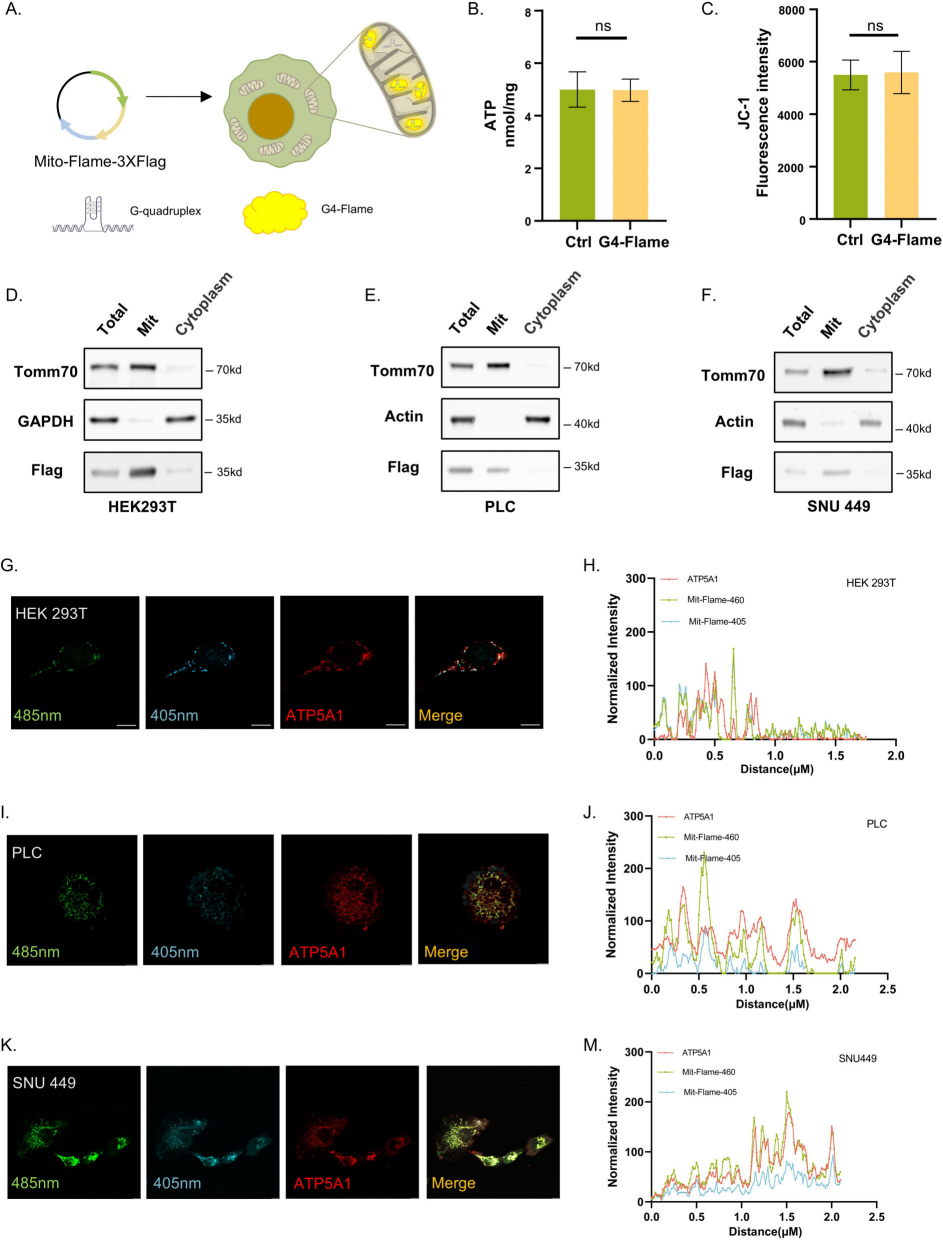
G4-Flame visualizes G4 Structures in cell mitochondria. (**A**) Schematic diagram of Mito-G4-Flame cell-line construction. Green: MTS sequence, yellow: G4-Flame sequence, and blue: 3× flag tag sequence. Arrows of different colors in the plasmid represent the corresponding functional elements shown in the diagram. (**B, C**). ATP levels and mitochondrial membrane potential (JC-1) in 293T cells transiently transfected with Mito-G4-Flame, compared with empty vector controls. No significant differences were observed between the two groups after transfection. (**D**–**F**) Subcellular fractionation assay validating Mito-G4-Flame mitochondrial targeting. WB probed with: anti-Tomm70 (mitochondrial marker), anti-GAPDH (cytoplasmic marker), anti-Actin (cytoplasmic marker), and anti-Flag (detecting G4-Flame). Results confirm exclusive mitochondrial localization of Mito-G4-Flame in stably transfected (D) HEK293T, (E) PLC, and (F) SNU 449 cells. Fluorescence images (**G, I**, and **K**) and co-localization analysis (**H, J**, and **M**) of HEK293T, PLC, and SNU 449 cells expressing Mito-G4-Flame (ex 485/405 nm, em 520 nm) costained with anti-ATP5A1 (mitochondrial marker). Scale bars: 10 µm. Data information: (B, C) are mean ± SD, unpaired *t*-test; ns, not significant.

The mitochondrial genome is a circular DNA molecule. Compared to nuclear DNA, mitochondrial DNA (mtDNA) is more prone to form G4 secondary structures due to more favorable sequence conditions. Crucially, the heavy strand (H strand) of mtDNA contains a significantly higher number of guanines relative to the light strand (L strand), violating the second parity rule and thereby increasing the likelihood of G4 formation. Despite this biological relevance, probes capable of visualizing mitochondrial G4-DNA in live cells remain scarce. To address this gap, we developed Mito-G4-Flame, a genetically encoded sensor for monitoring mitochondrial G4-DNA dynamics, by fusing a mitochondrial targeting sequence (MTS) to G4-Flame (Fig. 5A) and subsequently established a stable cell line expressing Mito-G4-Flame​ for sustained, noninvasive tracking of mtG4 dynamics. The probe was efficiently localized to mitochondria across multiple cell lines, as confirmed by subcellular fractionation and immunofluorescence analyses (Fig. 5D–F), and showed strong co-localization with the mitochondrial marker ATP5A1 (Fig. 5G–M). Importantly, Mito-G4-Flame did not significantly affect mitochondrial membrane potential (ΔΨm) or ATP production (Fig. 5B and C), supporting its suitability for noninvasive live-cell imaging.

**Figure 6.**
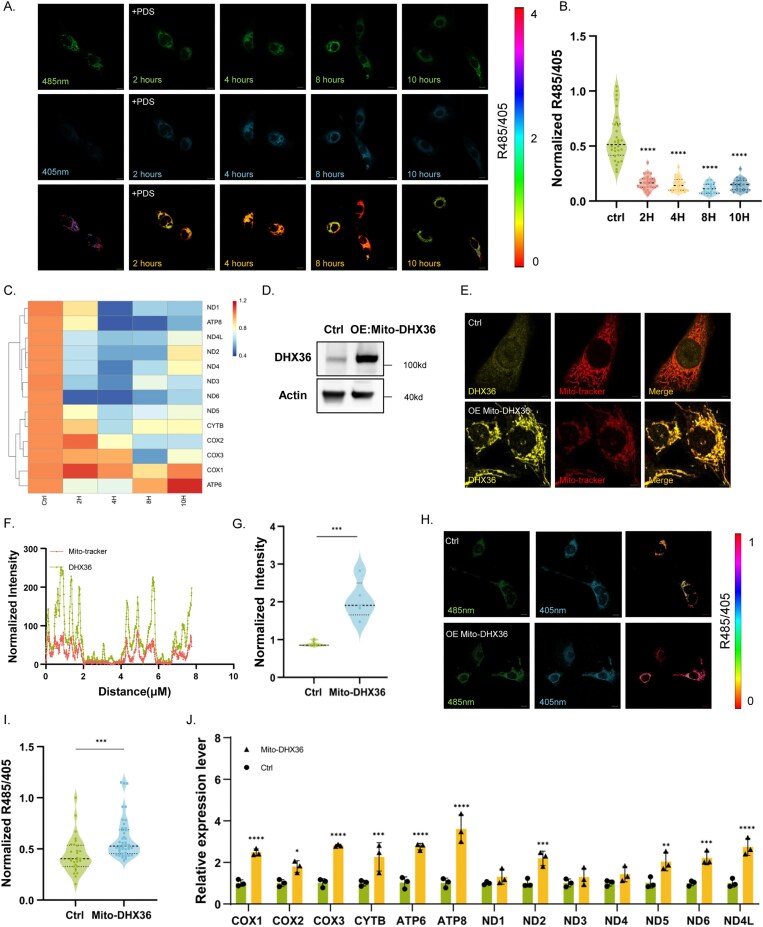
G4-Flame reveals mitochondrial G4-DNA suppresses mtDNA expression. Fluorescence images (**A**) and quantitative analysis (**B**) of SNU449 cells stably expressing Mito-G4-Flame. Cells were treated with 100 µM PDS for 2, 4, 8, and 10 h at 37 °C, showing a continuous decrease in R485/405 ratios over time. Scale bars: 10 µm. (**C**) PCR analysis of mitochondrial-encoded gene expression in SNU449 cells following PDS treatment across the indicated time points. Heatmap representation shows a time-dependent decrease in mRNA levels with increasing duration of PDS treatment. (**D**) WB analysis showing overexpression of mito-DHX36 in SNU449 cells stably expressing the construct. A strong DHX36 signal is observed, indicating robust expression in the mito-DHX36 stable cell line. Fluorescence images (**E**), colocalization analysis (**F**), and quantitative analysis (**G**) of SNU449 cells stably expressing mito-DHX36. DHX36 shows clear colocalization with mitochondria, and overexpression of mito-DHX36 leads to increased fluorescence intensity. Scale bars: 10 µm. Fluorescence images (**H**) and quantitative analysis (**I**) of SNU449 cells stably expressing Mito-G4-Flame with DHX36 overexpression. Overexpression of DHX36 in the stable cell line leads to an increase in R485/405 ratios. Scale bars: 10 µm. (**J**) qPCR analysis of mitochondrial-encoded gene expression in SNU449 cells stably expressing mito-DHX36. Overexpression of mito-DHX36 in the stable cell line led to increased expression of most mitochondrial-encoded genes. Data information: (J) are mean ± SD, (B) one-way ANOVA with Dunnett’s multiple comparisons test, (G and I) unpaired *t-*test, (J) two-way ANOVA with Dunnett’s multiple comparisons test; ns, not significant; *P < .05, **P < .01, ***P < .001, ****P < .0001.

To functionally validate the probe, cells were treated with the G4-stabilizing ligand PDS, which induced a time-dependent decrease in the 485/405 nm fluorescence ratio of Mito-G4-Flame (Fig. 6A and B), indicating enhanced G4-DNA accumulation. Corresponding analysis of mitochondrial-encoded gene expression revealed that the majority of these genes exhibited a time-dependent downregulation following PDS treatment (Fig. 6C), suggesting that binding of PDS to mitochondrial G4s (mtG4s) suppresses mtDNA transcription, accurately reflecting the experimental observation. To further elucidate the functional impact of mitochondrial G4-DNA, we sought to determine whether unwinding of G4-DNA by the resolvase DHX36 would produce the opposite effect to its stabilization by PDS. The mitochondrial localization of DHX36 was confirmed by western blot and immunofluorescence (Supplementary Fig. S6A–E). Overexpression of DHX36 resulted in enhanced transcription of most mitochondrial-encoded genes (Supplementary Fig. S6F and G). To exclude potential confounding effects from nuclear-localized DHX36, we constructed a mitochondrial-targeted DHX36 variant (mito-DHX36) to ensure specific expression within mitochondria (Fig. 6D–G). Upon induction of mito-DHX36 expression, we observed an increase in the R485/405 fluorescence ratio (Fig. 6H and I), indicating a reduction in G4-DNA levels. Consistent with the anticipated effect, this was accompanied by upregulation of most mitochondrial-encoded genes (Fig. 6J), supporting the conclusion that resolvase-mediated unwinding of G4-DNA promotes mitochondrial transcription.

In summary, Mito-G4-Flame serves as a versatile and noninvasive tool for real-time monitoring of mitochondrial G4-DNA dynamics, providing a new methodological foundation for investigating the role of G4 structures in mitochondrial biology and gene regulation.

### Clinical translation validation of G4-Flame for G4-DNA detection in tumor patients’ serum

**Figure 7.**
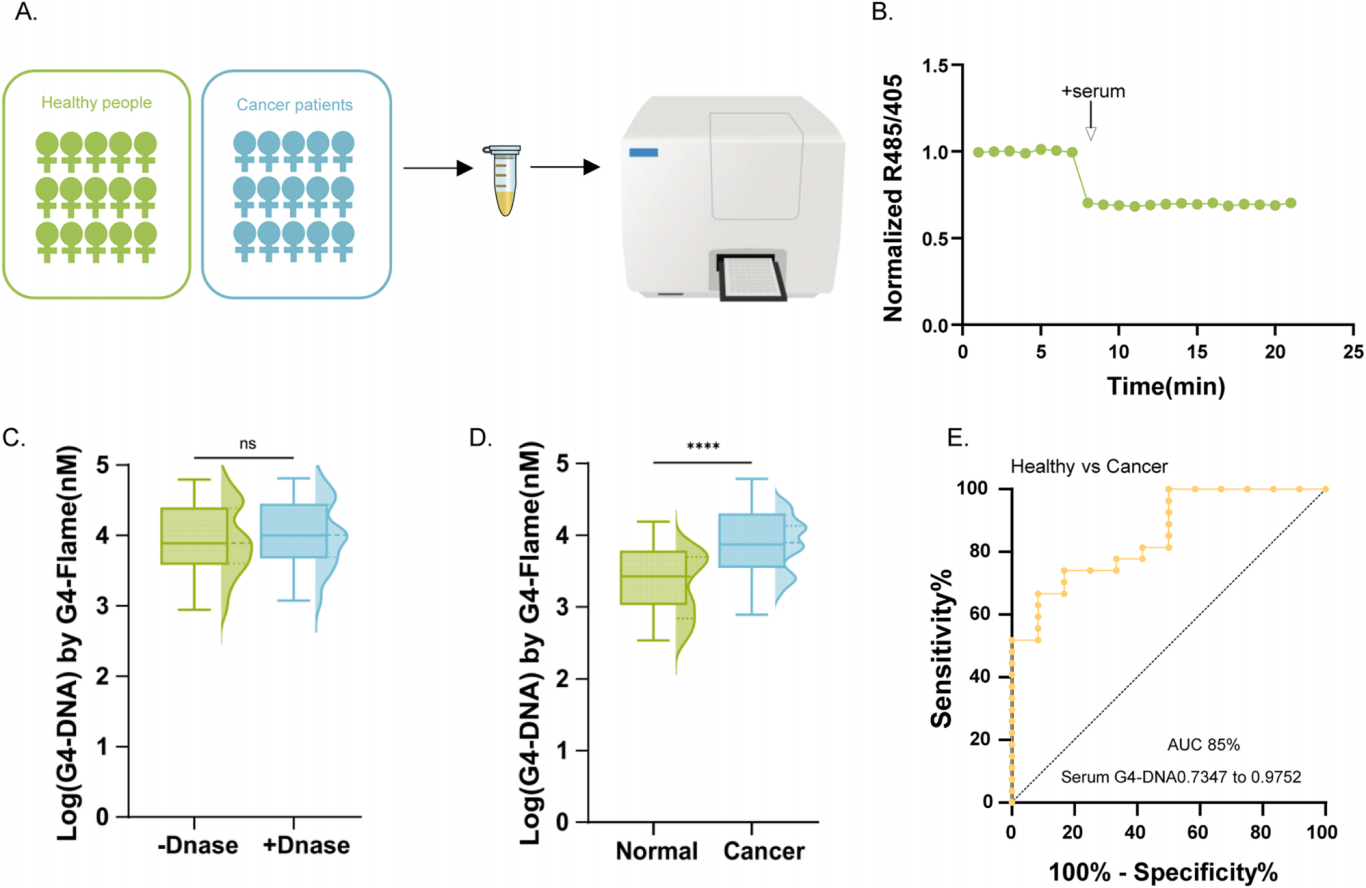
Convenient assay for G4-DNA in human serum of liver cancer patients. (**A**) Comprehensive workflow for G4-DNA quantification in human serum via G4-Flame fluorescence assay. (**B**) Kinetics of fluorescence response of the G4-Flame sensor upon addition of serum samples (*n* = 3). Upon addition of G4-Flame, the R485/405 ratio rapidly decreased and then stabilized. (**C**) G4-Flame quantification of serum G4-DNA in paired samples (*n* = 20) pre- and post-DNase treatment. Data are shown as combined violin and box plots, with no significant differences observed between the two conditions. (**D**) G4-Flame quantification of serum G4-DNA in tumor patients (*n* = 42) and healthy volunteers (*n* = 27). G4-Flame quantification of serum G4-DNA in tumor patients (*n* = 42) and healthy volunteers (*n* = 27). Data shown as combined violin and box plots. (**E**) ROC curves of patients with cancer compared with healthy controls. Data information: (B) are mean ± SD, (C) paired *t*-test, (D) unpaired *t*-test; ns, not significant; *P < .05, **P < .01, ***P < .001, ****P < .0001.

To evaluate the clinical application potential of G4-Flame, we examined its capability to detect G4-DNA in serum samples collected from cancer patients and healthy controls. A microscale detection system was established using 10 μl reactions in 384-well plates, with only 5 μl of serum required per test (Fig. 7A). Real-time fluorescence kinetic analysis revealed that G4-DNA in serum samples triggered characteristic fluorescence responses (changes in the R485/405 ratio) within 60 s, with signals stabilizing rapidly (Fig. 7B), demonstrating the sensor’s compatibility with clinical samples.

To further validate the stability of G4-DNA in bodily fluids, enzymatic resistance experiments (DNase I treatment) [39] were performed on 20 serum samples from tumor patients. The results showed no statistically significant differences in G4-DNA concentrations between treated and untreated groups (Fig. 7C). Additionally, quantitative analysis of expanded sample cohorts (42 tumor patients and 27 healthy controls) revealed that G4-DNA concentrations in cancer patients were significantly higher than those in healthy controls (Fig. 7D). Notably, although measured in a readily accessible biofluid rather than tissue, this elevation aligns with and functionally extends previous reports of increased G4-DNA levels in tumor tissues [40], underscoring the clinical relevance and practical advantage of serum-based detection. Further, receiver operating characteristic analysis demonstrated that serum G4-DNA exhibited significant discriminatory power in distinguishing cancer patients from healthy controls, with an area under the curve (AUC) value of 0.85 (Fig. 7E), further confirming the structural stability of G4-DNA and its possibility as a biomarker.

## Discussion

Traditional methods for detecting G4-DNA, including ThT, DAOTA-M2, SiR-PyPDS, and G4-DNA-specific antibodies such as BG4, have long been limited by several critical drawbacks. ThT, a widely used fluorescent dye, exhibits enhanced fluorescence upon binding to G4-DNA; however, it suffers from poor specificity and high sensitivity to environmental conditions, often yielding unreliable results in complex biological samples and limiting its applicability for precise *in vivo* studies [21, 22]. DAOTA-M2 enables the differentiation of G4-DNA from other nucleic acid structures via FLIM. Nonetheless, the high cost, operational complexity, and requirement for specialized instrumentation restrict its scalability and broader use [23, 24]. SiR-PyPDS, which combines an SiR fluorophore with the G4-specific ligand PDS, offers a more targeted approach for G4-DNA detection, but its multistep synthesis results in low yields (~30%) and challenging purification processes, limiting its feasibility for large-scale applications [25, 41]. BG4 antibodies, although highly specific for G4-DNA, rely on cell fixation, which not only perturbs the native folded conformation of G4-DNA and its protein interaction interfaces but also entirely precludes dynamic monitoring. Fixatives such as formaldehyde can induce G4 dissociation or abnormal rearrangements, effectively reducing experiments to static “snapshots of dead cells” and preventing real-time tracking of G4-DNA dynamics in living cells, including rapid assembly under stress or pathological disassembly. Consequently, BG4 is unsuitable for live-cell imaging or subcellular-level dynamic monitoring, limiting its utility for studying G4-DNA behavior *in vivo*. Although the nanobody SG4 [27] can achieve visualization of G4-DNA in cells through fusion with fluorescent proteins, its fluorescence readout is highly dependent on the expression level—since the expression level of the nanobody is susceptible to transfection efficiency, cell state, or metabolic fluctuations, the fluorescence signal intensity more likely reflects expression differences of the antibody itself rather than true abundance changes of G4-DNA, thus significantly increasing the difficulty of quantitative assessment. Therefore, the core value of SG4 mainly lies in providing spatial localization information of G4-DNA.

Taken together, these traditional approaches face substantial challenges in live-cell dynamic monitoring, high-resolution imaging, and scalability, underscoring the pressing need for novel probes capable of overcoming these limitations. In particular, there is an urgent demand for noninvasive, highly specific, and real-time detection tools that can transcend the static constraints of fixed samples, avoid nonspecific interference from chemical probes, and simultaneously achieve subcellular-level spatial resolution and millisecond-scale temporal sensitivity to capture the complete spatiotemporal dynamics of G4-DNA—from formation to functional execution—in living systems.

The development of G4-Flame represents a significant advance in nucleic acid structure detection, particularly for real-time monitoring of G4-DNA dynamics in biological contexts. By integrating the G4-DNA recognition domain RHAU23 with the cpYFP fluorescent protein scaffold, we have engineered a highly sensitive and specific molecular sensor capable of detecting G4-DNA at nanomolar concentrations. This dual-wavelength ratiometric fluorescence probe allows precise quantification via excitation at 405 and 485 nm, providing a robust platform for both *in vitro* and intracellular analyses.

One of the most compelling aspects of G4-Flame is its ability to maintain native G4-DNA structures without inducing conformational changes or disrupting integrity, as demonstrated by CD. This noninvasive binding mode, combined with its biocompatibility—evidenced by minimal impact on cell proliferation, gene expression, and mitochondrial function—makes it a promising tool for live-cell imaging and functional studies. The nuclear-localized variant (NLS–G4-Flame) has successfully revealed dynamic regulation of G4-DNA during the S phase of the cell cycle, aligning with established G4-DNA profiling techniques such as BG4 antibody staining and CUT&Tag sequencing [26]. These findings not only validate the accuracy of G4-Flame but also provide new insights into the temporal and spatial regulation of G4-DNA in mammalian cells.

Moreover, the mitochondrial-targeted version (Mito-G4-Flame) opens up a novel avenue for studying organellar G4-DNA, revealing for the first time the correlation between mitochondrial G4-DNA dynamics and the expression levels of mitochondrial-encoded genes. Although multiple lines of evidence have confirmed the existence of mitochondrial G4-DNA and its potential functional roles [42], effective tools for its direct visualization and dynamic monitoring in live cells have previously been lacking. Our study found that exogenous addition of PDS or overexpression of the G4-DNA helicase DHX36 led to significant reductions and elevations in mitochondrial G4-DNA levels, respectively, inducing corresponding changes in the expression levels of mitochondrial-encoded genes. This phenomenon suggests a potential regulatory mechanism linking G4-DNA stability to mitochondrial dysfunction, providing key insights into G4-DNA-associated diseases and mitochondrial genome instability.

From a clinical perspective, G4-Flame exhibits considerable translational promise. Using a serum-based detection assay, we demonstrated that G4-Flame enables rapid and reproducible quantification of G4-DNA in samples from cancer patients, clearly distinguishing them from healthy controls. With an AUC of 0.85, serum G4-DNA represents a promising biomarker for cancer diagnosis. The method’s minimal sample requirement and short assay time further support its potential in point-of-care testing and high-throughput screening. Although the mechanisms responsible for elevated serum G4-DNA levels in cancer patients remain incompletely understood, it is plausible that increased genomic instability and cell turnover in tumor tissues promote G4-DNA formation and subsequent release into the bloodstream—possibly through cell death or active secretion. Further studies are needed to clarify the origin and regulatory pathways of circulating G4-DNA.

Despite these significant advances, G4-Flame still has limitations that need to be addressed for broader applicability. First, as G4-Flame relies on fluorescent proteins, photobleaching remains a challenge for long-term dynamic monitoring, necessitating the use of anti-photobleaching agents or the development of more photostable variants. Second, the 405 nm excitation wavelength used in G4-Flame has limited tissue penetration, making it unsuitable for near-infrared *in vivo* imaging. Future improvements should focus on designing infrared-responsive fluorophores or fusing far-red fluorescent proteins to enable deeper tissue imaging and longitudinal tracking in living organisms.

In conclusion, G4-Flame serves as a powerful molecular tool for visualizing G4-DNA dynamics across multiple biological contexts—from biochemical assays to live-cell imaging and even clinical diagnostics. While current limitations suggest areas for improvement, addressing these challenges will expand the utility of G4-Flame from single-cell analysis to *in vivo* monitoring, ultimately enhancing our understanding of G4-DNA biology and accelerating its application in precision medicine and disease diagnostics.

## Supplementary Material

gkag179_Supplemental_Files

## Data Availability

The sequencing data have been deposited in the National Center for Biotechnology Information (NCBI) Gene Expression Omnibus (GEO) database under the accession number GSE314730.
