# Correction to ‘Development and application of G4-Flame as a visual biosensor for G4-DNA’

**DOI:** 10.1093/nar/gkag647

**Published:** 2026-06-23

This is a correction to: Ruyi Liu, Tao Wang, Chunxu Wang, Mingyou Xu, Boyu Chen, Jinyi Zhao, Wanxiang Xiong, Shixiang Pan, Zhao Ruan, Ningyuan Lu, Yuxin Zhang, Guang Yang, Fanzheng Meng, Yufeng Liu, Xuedan Sun, Lianxin Liu, Development and application of G4-Flame as a visual biosensor for G4-DNA, Nucleic Acids Research, Volume 54, Issue 6, 13 April 2026, gkag179, https://doi.org/10.1093/nar/gkag179

The authors wish to correct several errors in their article:

- Figure 3E: the y-axis labels are partly hidden.
- Figure 6C: the authors included the wrong heatmap.
- Figure 7B: one timepoint is missing.
- The data for Figure 7B in the supplementary files needs to be replaced.

Figures 3, 6 and 7 and the supplementary data have been replaced.

These changes do not affect the results, discussion and conclusions presented in the article. The published article has been updated.

