# Supplementary material for: Development and application of G4-Flame as a visual biosensor for G4-DNA
Source: Nucleic Acids Res. 2026 Mar 24;54(6):gkag179. doi: 10.1093/nar/gkag179 (PMC13010135; doi:10.1093/nar/gkag179)
Supplement: gkag179_Supplemental_Files [file gkag179_supplemental_files.zip › figureS-clear_new.pdf]

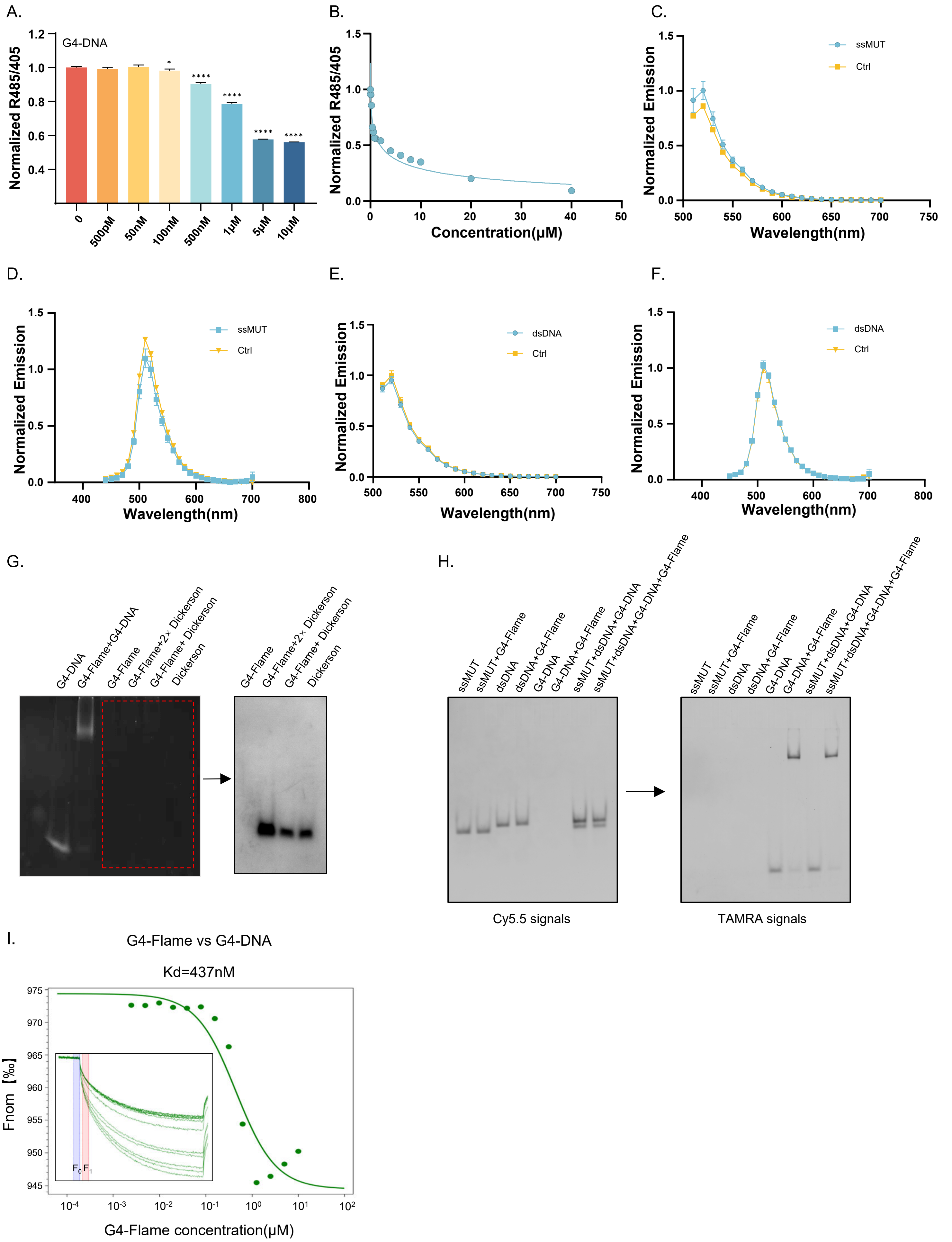
A.
G4-DNA
Normalized R485/405
0
500pM
50nM
100nM
500nM
1μM
5μM
10μM
*
****
B.
Normalized R485/405
Concentration(μM)
C.
ssMUT
Ctrl
Normalized Emission
Wavelength(nm)
D.
ssMUT
Ctrl
Normalized Emission
Wavelength(nm)
E.
dsDNA
Ctrl
Normalized Emission
Wavelength(nm)
F.
dsDNA
Ctrl
Normalized Emission
Wavelength(nm)
G.
G4-DNA
G4-Flame+G4-DNA
G4-Flame
G4-Flame+2× Dickerson
G4-Flame+ Dickerson
Dickerson
H.
ssMUT
ssMUT+G4-Flame
dsDNA
dsDNA+G4-Flame
G4-DNA
G4-DNA+G4-Flame
ssMUT+dsDNA+G4-DNA
ssMUT+dsDNA+G4-DNA+G4-Flame
Cy5.5 signals
TAMRA signals
I.
G4-Flame vs G4-DNA
Kd=437nM
Fnom [‰]
F0 F1
G4-Flame concentration(μM)

Figure S1. Spectral scanning analysis of various types of DNA

A. Fluorescence response (R485/405 ratio) of G4-Flame at the indicated concentration of G4-DNA (probe concentration: 2μM). Data are normalized to the initial value (n = 3).

B. Calibration Curve of G4-Flame with varying concentrations of G4-DNA.

C-F. Fluorescence intensities of G4-Flame with excitation wavelengths of 485 nm or 405 nm in the presence of ssMUT (C, D) and dsDNA (E, F).

G. Native PAGE analysis of G4-Flame specificity for G4-DNA versus dsDNA. 10 or 20 μM Dickerson was incubated 2 μM G4-Flame. Signals of Dickerson was detected by staining with methylene blue.

H. Competition assay of G4-Flame towards ssMUT, dsDNA and G4-DNA. Lane 1-6 included separated titration with G4-Flame toward ssMUT/dsDNA/G4-DNA. ssMUT and dsDNA were cyc5.5-labeled while G4-DNA was TAMRA labeled. Lane 7 and 8 showed the result of the competition assay. All three types of DNA were mixed together in samples loaded to these 2 lanes. G4-Flame was added in the sample loaded to Lane 8. The G4-Flame displayed obvious binding to G4-DNA only.

I. In vitro binding between G4-Flame and G4-DNA. The binding affinity between G4-Flame and G4-DNA was measured by microscale thermophoresis (MST). Inset, thermophoretic movement of fluorescently labeled proteins. $F_{norm} = F_1/F_0$ (F_{norm}: normalized fluorescence; F_1: fluorescence after thermodiffusion; F_0: initial fluorescence or fluorescence after T-jump). Kd, dissociation constant.

Data information: (A-F) are mean ± SD; (A) one-way ANOVA with Dunnett's multiple comparisons test; ns, not significant; *p < 0.05, **p < 0.01, ***p < 0.001, ****p < 0.0001.

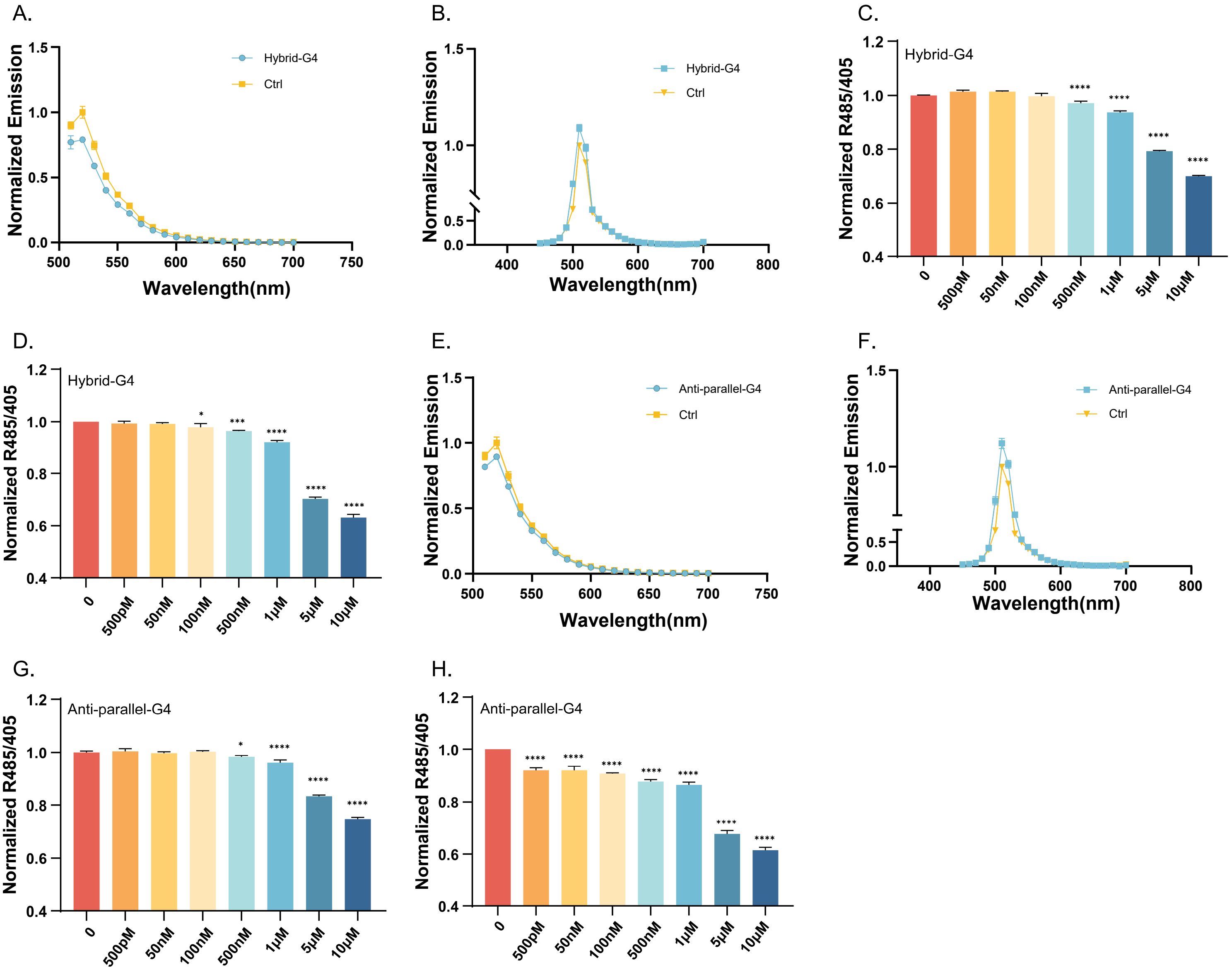

Figure S2. Spectral scanning of different types of G4-DNA structures

A-B. Fluorescence intensities of G4-Flame with excitation wavelengths of 485 nm or 405 nm in the presence of Hybrid-G4.

C. Fluorescence response (R485/405 ratio) of G4-Flame at the indicated concentration of Hybrid-G4 (probe concentration: 2μM). Data are normalized to the initial value (n = 3).

D. Fluorescence response (R485/405 ratio) of G4-Flame at the indicated concentration of Hybrid-G4 (probe concentration: 500nM). Data are normalized to the initial value (n = 3).

E-F. Fluorescence intensities of G4-Flame with excitation wavelengths of 485 nm or 405 nm in the presence of anti-parallel-G4.

G. Fluorescence response (R485/405 ratio) of G4-Flame at the indicated concentration of anti-parallel-G4 (probe concentration: 2μM). Data are normalized to the initial value (n = 3).

H. Fluorescence response (R485/405 ratio) of G4-Flame at the indicated concentration of anti-parallel-G4 (probe concentration: 500nM). Data are normalized to the initial value (n = 3).

Data information: (A-H) are mean ± SD; (C, D, G, and H) one-way ANOVA with Dunnett's multiple comparisons test; ns, not significant; *p < 0.05, **p < 0.01, ***p < 0.001, ****p < 0.0001.

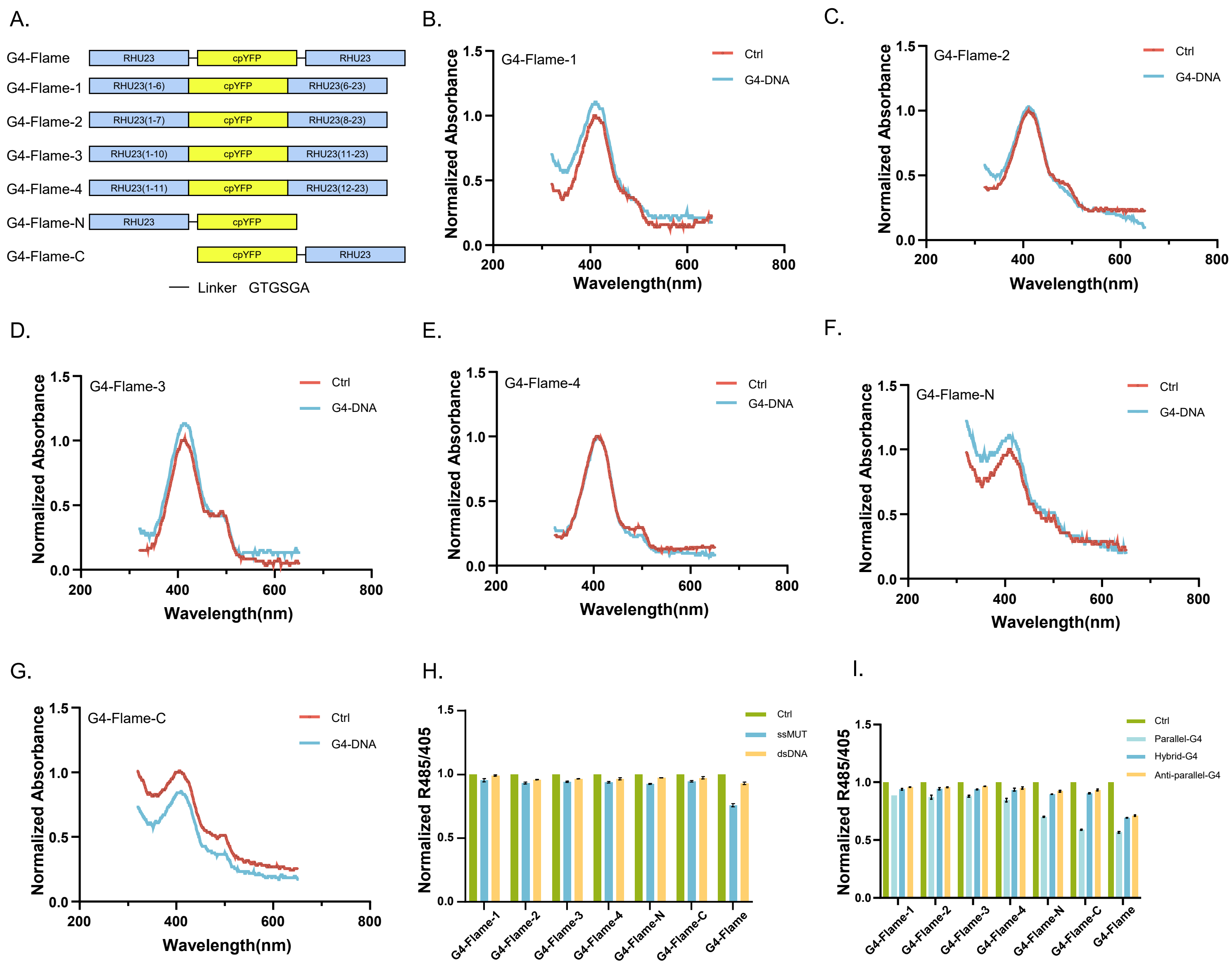

Figure S3. G4-Flame Probe Variants and Their Fluorescence Responses to Different DNA Types

A.Schematic model for seven RUAH23 and cpYFP chimeras.

B-G. The absorption spectra of G4-Flame probe variants (10μM) under conditions with and without G4-DNA (10μM)**.** (B) G4-Flame-1, (C) G4-Flame-2, (D) G4-Flame-3, (E) G4-Flame-4, (F) G4-Flame-N, (G) G4-Flame-C.

H. Fluorescence responses of seven chimeric proteins in the presence of 10 μM different types of DNA.

I. Fluorescence responses of seven chimeric proteins in the presence of 10 μM different types of G4-DNA

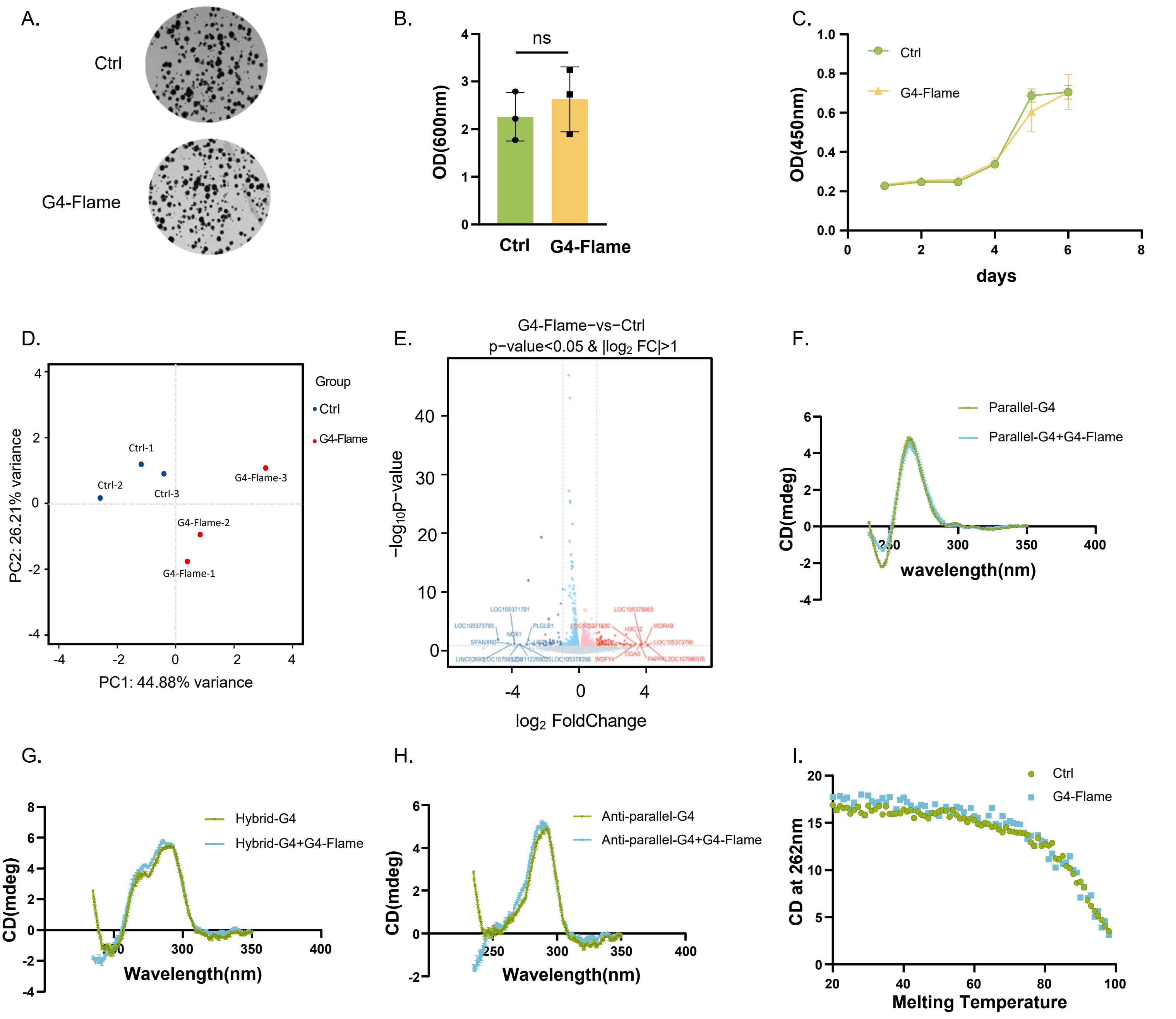

Figure S4. G4-Flame exhibits reliable biocompatibility

A-B. Colony formation (A) and quantification analysis (B) of HCCLM3 cells stably transfected with G4-Flame (n=3).

C. Growth curve analysis of HCCLM3 cells stably transfected with G4-Flame versus control cells by CCK-8 assay.

D. PCA analysis of transcriptome data from HEK293T cells expressing G4-Flame and control groups.

E. Volcano plot analysis of differentially expressed genes between HEK293T cells expressing G4-Flame and control groups. Red: Significant Up; Pink: Up; Gray: Non-significant dark; Blue: Significant Down; Light blue: Down. $p<0.05$, $|log2FC|>1$.

F-H. CD spectral analysis of purified G4-Flame with (F)parallel-G4, (G)Hybrid-G4, (H)Anti-parallel-G4. (n=3).

I. Thermal stability of G4-DNA in the presence and absence of G4-Flame, as determined by CD-Tm analysis. The melting temperature (Tm) reflects the temperature at which half of the G4-DNA molecules undergo unfolding.

Data information: (B, C) are mean ± SD; (B) paired t test; ns, not significant.

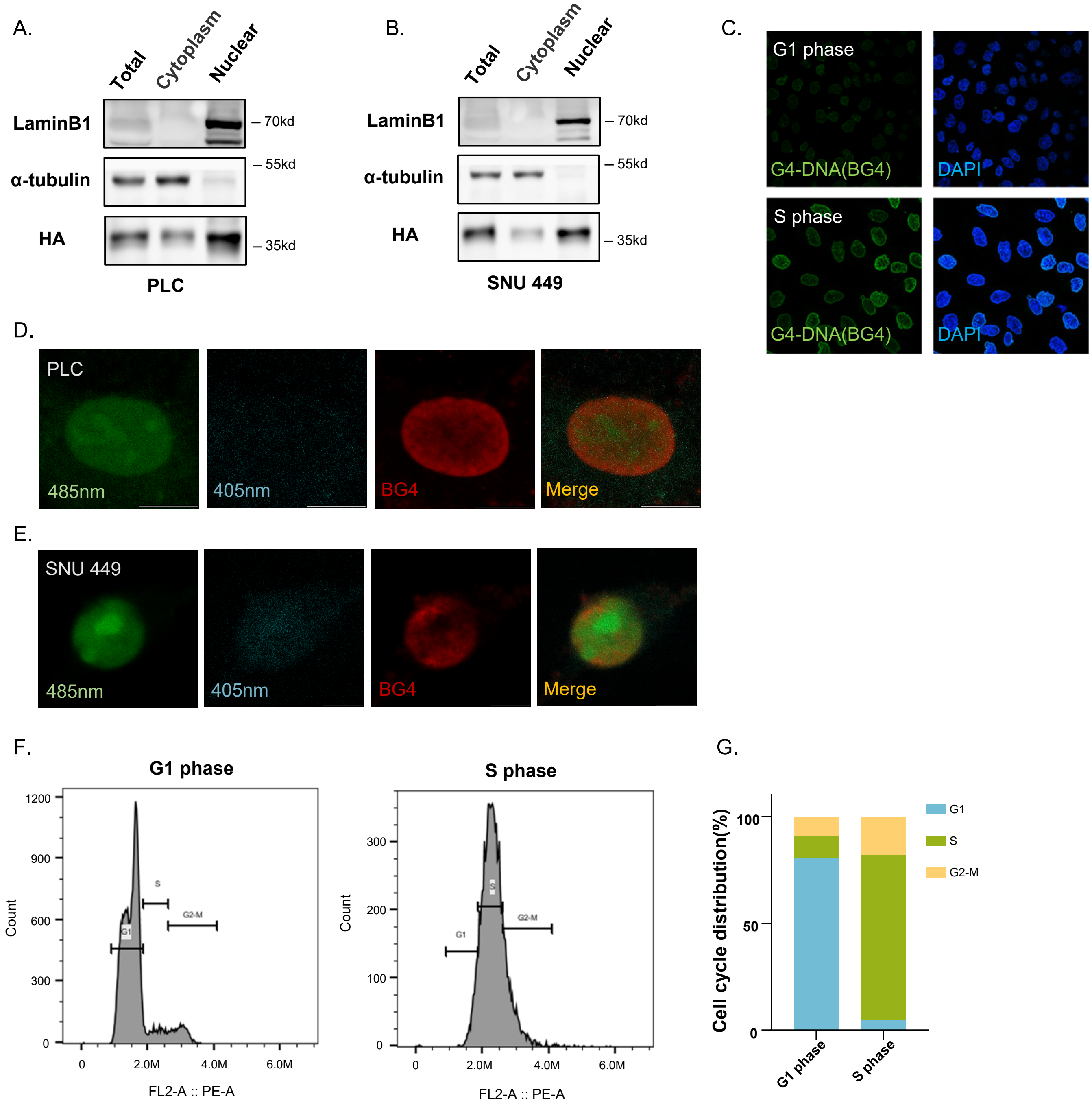

Figure S5. Construction of NLS-G4-Flame Cell Line

A-B. Subcellular fractionation assay validating NLS-G4-Flame nuclear targeting. Western blots probed with: anti-HA (detecting G4-Flame) and anti-Lamin B1 (nuclear marker); anti-α-tubulin (cytoplasmic marker). Results confirm exclusive nuclear localization of HA-G4-Flame in stably transfected PLC and SNU 449 cells.

C. Confocal microscopy images of G4-DNA structures in SNU 449 cells during G1 and S phases, immunostained with BG4 antibody. Scale bars,10 µm.

D-E. Confocal microscopy images of PLC and SNU 449 cell lines expressing NLS-G4-Flame, co-labeled with BG4 (G4-DNA antibody). Scale bars, 10 µm.

F. Flow cytometry histograms illustrating DNA content distribution in G1-phase–enriched and S-phase–enriched cells. The *x*-axis represents the fluorescence intensity of FL2-A (PE-A), which correlates with cellular DNA content after propidium iodide (PI) staining. The *y*-axis indicates the number of cells (count). The left panel corresponds to G1-phase–enriched cells, and the right panel corresponds to S-phase–enriched cells. Regions labeled “G1”, “S”, and “G2-M” identify cell populations in the respective cell cycle phases.

G. Bar graph depicting the percentage distribution of cells in G1, S, and G2-M phases for G1-phase–enriched and S-phase–enriched cells. Blue: G1; green: S; and yellow: G2-M.

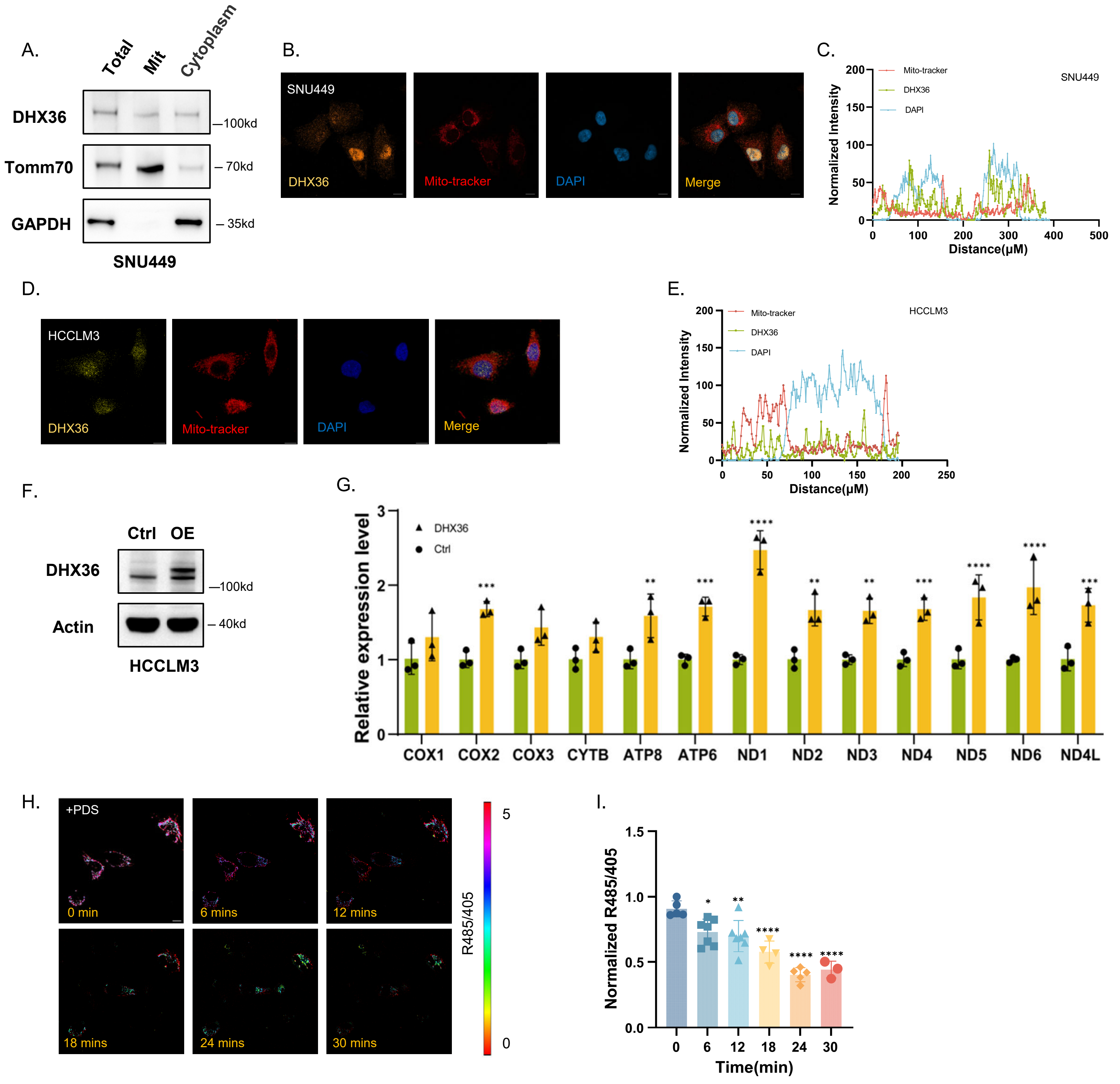

Figure S6. G4-Flame reveals the regulatory role of DHX36 in mitochondrial gene expression

A. WB analysis of endogenous DHX36 expression in mitochondria of SNU 449 cells.

B-C. Fluorescence images (B) and co-localization analysis © of the basal signals of DHX36 in SNU 449 cells. Scale bars, 10 μm.

D-E. Fluorescence images (D) and co-localization analysis € of the basal signals of DHX36 in HCCLM3 cells. Scale bars, 10 μm.

F. WB analysis showing overexpression of DHX36 in HCCLM3 cells.

G. Qpcr analysis of mitochondrial-encoded gene expression in HCCLM3 cells following DHX36 overexpression.

H-I. Live-cell fluorescence images (H) and quantitative analysis (I) of SNU 449 cells expressing Mito-G4-Flame. Cells were treated with 20μM PDS at 37°C. Scale bars, 10 μm.

Data information: (G, I) are mean ± SD; (G) two-way ANOVA with Dunnett's multiple comparisons test; ns, not significant; (I) one-way ANOVA with Dunnett's multiple comparisons test; ns, not significant; *p < 0.05, **p < 0.01, ***p < 0.001, ****p < 0.0001.
